# The Earth’s Magnetosphere: A Systems Science Overview and Assessment

**DOI:** 10.1007/s10712-018-9487-x

**Published:** 2018-07-20

**Authors:** Joseph E. Borovsky, Juan Alejandro Valdivia

**Affiliations:** 1 Center for Space Plasma Physics, Space Science Institute, Boulder, CO 80301 USA; 2 Departmento de Fisica, Facultad de Ciencias, Universidad de Chile, 7800003 Santiago, Chile

**Keywords:** Magnetosphere, Complex systems, Systems science, Emergence, Coherent structure, Radiation belt

## Abstract

A systems science examination of the Earth’s fully interconnected dynamic magnetosphere is presented. Here the magnetospheric system (a.k.a. the magnetosphere–ionosphere–thermosphere system) is considered to be comprised of 14 interconnected subsystems, where each subsystem is a characteristic particle population: 12 of those particle populations are plasmas and two (the atmosphere and the hydrogen geocorona) are neutrals. For the magnetospheric system, an assessment is made of the applicability of several system descriptors, such as adaptive, nonlinear, dissipative, interdependent, open, irreversible, and complex. The 14 subsystems of the magnetospheric system are cataloged and described, and the various types of magnetospheric waves that couple the behaviors of the subsystems to each other are explained. This yields a roadmap of the connectivity of the magnetospheric system. Various forms of magnetospheric activity beyond geomagnetic activity are reviewed, and four examples of emergent phenomena in the Earth’s magnetosphere are presented. Prior systems science investigations of the solar-wind-driven magnetospheric system are discussed: up to the present these investigations have not accounted for the full interconnectedness of the system. This overview and assessment of the Earth’s magnetosphere hopes to facilitate (1) future global systems science studies that involve the entire interconnected magnetospheric system with its diverse time and spatial scales and (2) connections of magnetospheric systems science with the broader Earth systems science.

## Introduction

The purposes of this review are (1) to provide an overview of the magnetospheric system (i.e., its subsystems and how the subsystems interact), to show how the system is driven by the solar wind, and how it reacts to the driving; (2) to describe the various phenomena that collectively make up magnetospheric activity; (3) to assess the system properties of the magnetosphere; and (4) to briefly discuss systems science studies of the magnetosphere that have been performed and that have yet to be performed. The motivation for this overview is the statement of Lin et al. (2013). “Thus, when hoping to understand the behaviors of a complex system, one needs to analyze not only how different components work together to form the behaviors of the whole system, but also the behaviors of the individual parts. Without deep and specific comprehension of the behaviors of the individual parts, there will be no way to capture the behaviors of the complex system.” Systems science studies of the magnetosphere have been ongoing for a few decades, mostly focused on the analysis of a single measure of magnetospheric activity. This review will encourage more such studies and will try to lay a foundation for future studies dealing with the global nature of the magnetospheric system and its interaction with the Earth system. At present, such global studies do not exist.

This paper is aimed primarily at four types of readers: (1) a systems scientist who wants to think about this complicated, rich, important system, (2) an Earth systems scientist who wants to know how the magnetospheric system operates and influences the atmosphere and atmospheric electricity, (3) a beginning magnetospheric researcher who could utilize an overview of how the magnetosphere operates, and (4) a magnetospheric expert interesting in applying systems science to the magnetosphere.

An understanding of the Earth’s magnetosphere is important for a number of reasons. (1) It is the environment that the Earth exists in and so it is natural to want to understand that environment. (2) The Earth’s magnetosphere is also the closest astrophysical system that can be studied, and it is a very-well-measured system. (3) The activation of the Earth’s magnetosphere by the solar wind gives rise to “space weather,” which poses real dangers to astronauts, to the operation of spacecraft, and to electrical power grids at high latitudes, among others: based on magnetospheric physics, there is a substantial research effort to understand and forecast space weather. (4) The precipitation of energetic electrons from the magnetosphere into the atmosphere affects the chemistry and degree of ionization of the middle atmosphere and ionospheric Joule heating from the magnetosphere affects thermospheric temperatures, densities, and winds, and therefore, the magnetosphere plays a role in Earth systems science. (5) The magnetosphere provides a unique laboratory for plasma physics, especially for the all-important process of magnetic-field-line reconnection and also for the physics of collisionless shocks.

This paper is organized as follows: In Sect. 2, an overview of the Earth’s magnetospheric system as driven by the time-dependent solar wind is given. In Sect. 3, the morphology and geographic regions of the magnetosphere are discussed. In Sect. 4, some physical processes that are important for the operation of the magnetospheric system are explained. The diverse particle populations of the magnetosphere, considered here as the subsystems of the magnetospheric system, are cataloged and discussed in Sect. 5. In Sect. 6, the various types of waves in the magnetosphere are cataloged and discussed: plasma waves are the manner by which the various subsystems of the magnetosphere interact with each other. Section 7 discusses the various types of activity in the magnetospheric system, i.e., the many ways that the system responds to driving by the solar wind. Section 8 discusses emergent phenomena in the magnetospheric system when it is driven by the solar wind. Section 9 discusses a number of system descriptors (adjectives) and how they apply to the magnetospheric system, the most fundamental of which being the question of whether the Earth’s magnetosphere is a “complex system.” Section 10 briefly reviews systems science research that has been performed on the Earth’s magnetosphere as driven by the solar wind and discusses how magnetospheric systems science fits into Earth systems science. Section 11 contains discussions about things that are not yet known about the operation of the Earth’s magnetosphere.

## Overview of the Earth’s Magnetosphere

The interaction of the supersonic solar wind with the Earth’s dipole magnetic field is surprisingly complicated. About 5 orders of magnitude of spatial scales are involved in the global behavior of the magnetospheric system and timescales from seconds for the auroral pulsations (Yamamoto 1988), to several minutes for the reaction of the global magnetosphere to solar-wind pressure changes (Boudouridis et al. 2011), to several days for the intensification of the electron radiation belt (Balikhin et al. 2011), to years for the decay of relativistic electrons (Stassinopoulos and Verzariu 1971). Plasma-physical length scales vary from a Debye length of 0.4 cm in the ionosphere to ion gyroradii of 1000 km in the magnetotail and in the ion radiation belt; plasma-physical timescales associated with wave substructure can also be very short.

**Fig. 1:**
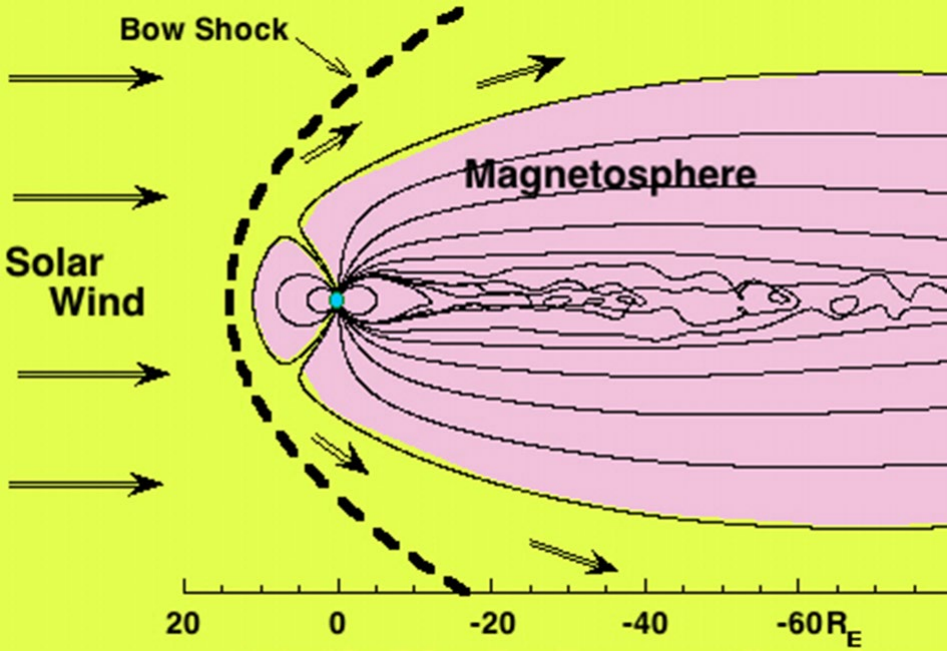
A depiction of the Earth (blue) and its magnetosphere (shaded in pink) bathed in solar-wind plasma (yellow). The thin black lines are magnetic-field lines. The solar-wind plasma is flowing from left to right

**Fig. 2:**
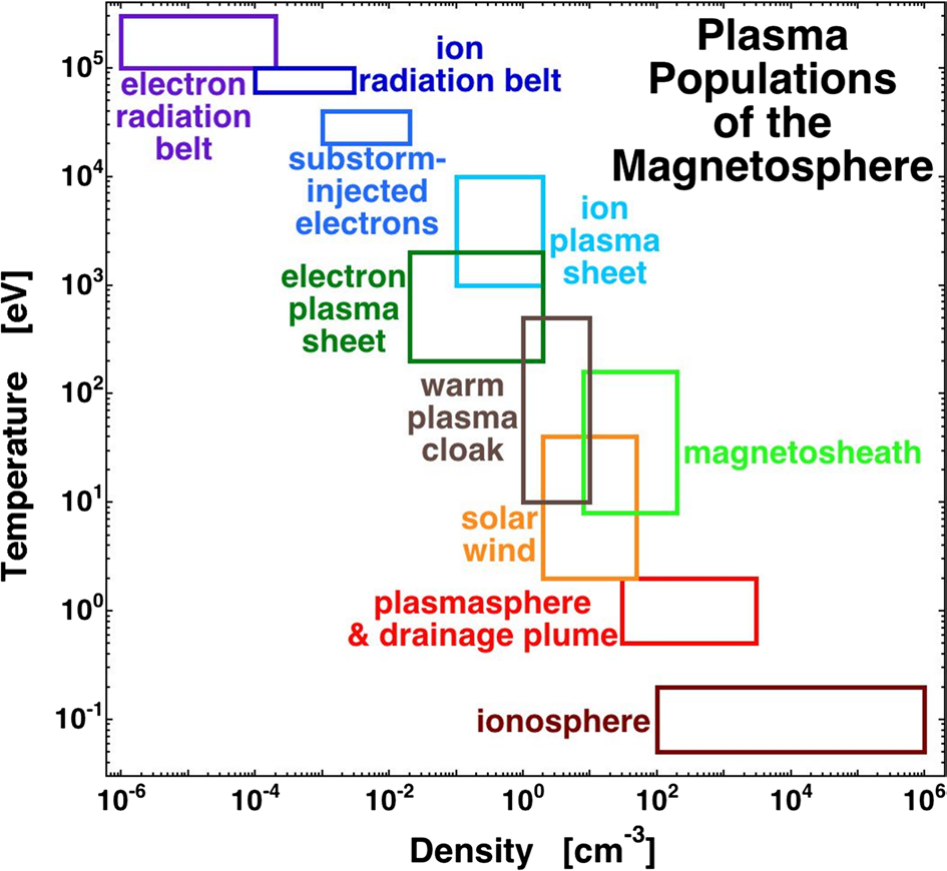
The ranges of temperature (vertical) and number density (horizontal axis) of several of the plasmas of the Earth’s magnetosphere are plotted. The values for the ion and electron radiation belt pertain to values seen at geosynchronous orbit: closer to the Earth those populations are hotter

The Earth’s magnetosphere is the spatial domain of the magnetic-field lines that connect to the Earth. This is sketched in Fig. 1, with the magnetosphere shaded in pink. Note the spatial scale indicated at the bottom in units of Earth radii *R*_E_, with 1 *R*_E _= 6378 km. A magnetic field has the property that it holds charged particles (ions and electrons), and when ions and electrons build up in the magnetic field to a sufficient density, they become a gas (called a plasma) that exhibits electromagnetic collective behavior such as plasma waves. The plasma can be described as having a number density *n* and a temperature *T*, and it can have a vector flow velocity *v*. There are multiple plasma populations with diverse properties that build up and evolve in the Earth’s magnetosphere (cf. Fig. 2). The different plasmas interact with each other via different types of electromagnetic plasma waves that grow and damp throughout the magnetosphere.

The evolution and activation of the Earth’s magnetosphere is driven by the solar wind, which is a supersonic magnetized plasma wind from the Sun that fills the solar system (Richardson 2013). Typical speeds of the solar wind are 300–800 km/s. Without the solar wind, the Earth’s magnetosphere would be a magnetic dipole. However, the magnetosphere is distorted by the solar wind (see Fig. 1): it is compressed on the sunward side (dayside) and pulled out into a long tail on the anti-sunward side (nightside). The magnetosphere being driven by the solar wind means that the solar wind transfers plasma and energy into the magnetosphere, and very importantly, it sets up a global convection of plasma in the magnetosphere. This convection is important for transporting plasmas inside the magnetosphere, for energizing plasma, for driving waves in the plasmas, and for exhausting plasma out of the magnetosphere. At Earth, the properties of the solar wind vary with time owing (1) to the spotty sources of wind on the Sun’s surface and the 27-day rotation of the Sun as seen by Earth and (2) to the fine-scale spatial structure of the solar-wind plasma; this means that the strength and details of the driving of the magnetosphere vary with time.

The Earth’s magnetosphere has been measured by spacecraft instrumentation since the 1960s (Stern 1996). Much of the spacecraft data is publically available from the NASA Space Physics Data Facility at https://cdaweb.sci.gsfc.nasa.gov/index.html/, and solar-wind measurements at Earth from 1963 to the present are available at https://omniweb.gsfc.nasa.gov. Sections 3–7 will explain several aspects of the magnetospheric system. There are also excellent tutorials about the Earth’s magnetosphere (Lyon 2000; Otto 2005; Siscoe 2011; Eastwood et al. 2015) and excellent textbooks (Egeland et al. 1973; Jursa 1985; Hargreaves 1992; Kivelson and Russell 1995; Gombosi 1998).

The magnetosphere exhibits several classic systems aspects (cf. Sect. 10). (1) The magnetosphere is comprised of multiple interacting subsystems (the various plasmas). (2) The physical interactions within the system are multiscale, both local and global: small scale is ~ 1 km (e.g., the reconnection diffusion region), global is 3 × 10^5^ km, with the global morphology of the magnetosphere at times being strongly affected by a single reconnection diffusion region set up either at the front of the magnetosphere or in the magnetotail. (3) The magnetosphere has a region that is turbulent and the magnetosphere exhibits some behavior that appears chaotic. (4) It will be shown in Sect. 8 that the magnetosphere exhibits emergent phenomena, so it could be argued that “the magnetosphere is greater than the sum of its parts.”

## The Morphology of the Magnetosphere

The solar wind distorts the dipole magnetic field of the Earth, compressing the dayside and drawing the nightside out into a magnetotail. Note from Ampere’s law of magnetism that every distortion of the magnetic field is associated with an electrical current. There are several current systems in the magnetosphere (Liemohn et al. 2015), but they will not be discussed here.

In Table 1, the names of several geographic regions of the Earth’s magnetosphere are listed. The first location is the *bow shock* (Fairfield 1971; see Fig. 1). Because the solar wind is flowing supersonically at the Earth’s magnetosphere, which is an obstacle to the flow, a shock wave forms in the flow converting the supersonic flow into a subsonic flow. The bow shock heats and compresses the solar-wind plasma and deflects the plasma flow around the magnetospheric obstacle.

**Table 1:** Locations in the magnetospheric system

Location	Importance
Bow shock	Processes cool solar wind into hot magnetosheath
Magnetopause	Outer boundary of magnetosphere
	Site of dayside reconnection
	A location of plasma entry into magnetosphere
Dipolar region	Traps plasma and energetic charged particles
Magnetotail	Reservoir of magnetic flux and energy
	Globally unstable at times: energy for substorms
Ionosphere and thermosphere	A source of plasma for magnetosphere
	An absorber of magnetospheric charged particles
Cusps	A location of plasma entry into magnetosphere
Auroral zone	A region of energy transfer from the magnetosphere to the ionosphere
Geosynchronous orbit	Populated with spacecraft, *r* = 6.6 *R*_E_
Lunar orbit	Orbit of the moon, *r* = 60 *R*_E_
L1 Lagrangian point	Location of upstream solar-wind monitors, 235 *R*_E_ toward the Sun

The second region in Table 1 is the *magnetopause* (Safrankova et al. 2002). This is the outer boundary of the magnetosphere, the end of the domain wherein magnetic-field lines connect to the Earth. In Fig. 1, this is the boundary between the pink and yellow regions. Typically there are electrical currents flowing on the magnetopause. Just inside of the magnetopause there are “boundary layers”: at low latitudes the boundary layer consists of a plasma called the low-latitude boundary layer (see Sect. 5) and at high latitudes the boundary layer consists of a plasma known as the mantle (see Sect. 5).

The third location in Table 1 is the *dipolar region* or inner magnetosphere (Olson and Pfitzer 1974) of the magnetosphere near the Earth. The strength of a dipole magnetic field falls off as 1/*r*^3^, where *r* is the radial distance, so that at small distances *r* close to the Earth the magnetic field is strong and the distortions caused by the solar wind are not effective. The Earth rotates with a 24-h period, and the portion of the dipolar magnetosphere that is closest to the Earth corotates with the Earth, the cooler plasmas in that near-Earth region having a 24-h circulation period. (The corotation is caused by the tendency of the ionosphere to be collisionally coupled to the atmosphere (Rees 1989), which rotates with the Earth.) The radiation belts (see Sect. 5) reside in the dipolar region, as does the plasmasphere (see Sect. 5).

The fourth region in Table 1 is the *magnetotail* (Nishida 2000) on the nightside of the Earth. It is a very long (100’s of *R*_E_), cylindrical volume of magnetic-field lines connected to the Earth, with solar-wind plasma flowing away from the Sun outside of the magnetotail. Within the magnetotail two important hot plasmas reside: the ion plasma sheet and the electron plasma sheet. The global pattern of convection in the magnetosphere is from nightside to dayside (opposite to the direction of the solar-wind flow), and the near-Earth portion of the magnetotail is a conduit for the delivery of hot plasma into the dipolar magnetosphere from the nightside. The magnetotail is a reservoir of magnetic energy that powers several magnetospheric processes. The magnetotail is subject to global instabilities that produce “substorms,” which are surges of Earthward convection that occur on average a few times per day: accompanying the occurrence of a substorm, a magnetized plasmoid is ejected down the magnetotail to be lost from the Earth.

The *ionosphere* and the *thermosphere*. The thermosphere is the upper region (above ~ 85 km) of the neutral atmosphere. The ionosphere is the ionized portion of the thermosphere.

The *cusps* (Sandahl 2003) are features in the magnetic field of the magnetosphere close to the northern and southern poles of the Earth’s dipole where solar-wind plasma can penetrate deep into the magnetosphere along the magnetic-field lines. In Fig. 1, the cusps can be seen as the two intrusions of yellow solar-wind plasma into the pink magnetosphere.

The *auroral zone* (Feldstein and Galperin 1985) is a ring around the northern polar region of the Earth and a ring around the southern polar region where magnetospheric electrons and ions impact the upper atmosphere to produce visible airglow. Most aurora are produced by electron impact from the electron plasma sheet. A weaker airglow is produced by proton impact (from the ion plasma sheet) on the atmosphere at latitudes slightly lower than the electron aurora. Aurora represent a large amount of power from the magnetosphere that is dissipated in the atmosphere and ionosphere.

Table 1 points out three other locations important to the magnetosphere. *Geosynchronous orbit* (*r* = 6.6 *R*_E_) is the location where a satellite has a gravitational orbit that has a 24-h period and hence where orbiting objects corotate with the surface of the Earth; geosynchronous orbit is heavily populated with communications satellites. *Lunar orbit* (*r* ≈ 60 *R*_E_) is the location of the Moon’s orbit, with the Moon crossing through the Earth’s magnetotail when the Moon is full (cf. Fig. 1). The *first Lagrangian point L1* is 235 *R*_E_ upstream of the Earth along the Sun–Earth line: it is a quasi-stable gravitational-orbit location that is used to place solar-wind-monitoring spacecraft upstream of the Earth. The solar-wind plasma that passes a spacecraft at L1 hits the Earth 30–60 min later (Weimer et al. 2003).

## Important Physical Processes

There are several physical processes that are important to the operation of the Earth’s magnetosphere.

*Magnetic-field-line reconnection* (Birn and Priest 2009) is an extremely important process for the magnetosphere. Reconnection changes the magnetic connection between two magnetized plasmas. There are two major sites of reconnection. (1) On the dayside magnetopause reconnection magnetically connects the moving solar-wind plasma to the magnetosphere. This magnetic connection allows the solar wind to couple to the magnetosphere, transferring plasma, magnetic field, momentum, and energy into the magnetosphere. The amount of reconnection (the reconnection rate) governs the amount of solar-wind/magnetosphere coupling; hence, it governs most of the driving of the magnetosphere by the solar wind. (2) In the magnetotail, reconnection changes the magnetic morphology of the tail, driving magnetospheric global convection and allowing the tail to move to a lower energy state, converting magnetic energy into flows, heating, and particle energization.

Several concepts relate to the orbits of charged particles in a magnetic field (Falthammar 1973). In a vacuum, charged particles move around a magnetic-field line in a spiral orbit, being free to move along the field line but performing circular (cyclotron) motion perpendicular to the field line. In a dipole magnetic field, charged particles perform bounce motion as they spiral, where there is a “*mirror*” *force* along the field line that pushes the particle toward the equator of the dipole, acting to keep it away from the Earth. For charged particles that have velocity vectors nearly parallel to the field line, this mirror force is ineffective and the charged particle can reach the Earth’s atmosphere, where it is lost from the magnetosphere. Furthermore, energetic particles are subject to *magnetic drifts*, wherein the morphology of the magnetic field allows the particles to drift across the magnetic-field-line direction.

Cold plasma is transported in the magnetosphere differently from hot plasma. Cold plasma follows the convection of magnetic-field lines. The *global magnetospheric convection* of magnetic-field lines (Dungey 1961) is from the magnetotail sunward through the dipolar portion of the magnetosphere to the dayside magnetopause; hence, cooler plasmas follow this magnetic convection and they exit the magnetosphere at the dayside magnetopause. Note, however, very close to the earth there is a zone where the cold plasma corotates with the earth and is trapped. The convection of hot plasma tends to be dominated by the shape of the magnetic-field lines. The ions and electrons of the hottest plasmas tend to make circular orbits around the Earth, with the positive ions moving westward around the Earth and the negative electrons moving eastward around the Earth.

*Plasma waves* are electromagnetic fluctuations in the plasma. In a magnetized plasma, there is a “zoo” of different possible wave types (Walker 1993). If there is a free energy source (which there often is in the magnetosphere), the waves in the plasma can grow in amplitude (Gary 1993). For example, particular consideration can be given to the thermal and nonthermal properties of the particle velocity distribution functions which can have profound effects in wave propagation and generation, as well as thermal anisotropy that can provide a natural surge of free energy for the growth (Valdivia et al. 2016).

*Wave-particle interactions* (Tsurutani and Lakhina 1997) occur because the charged particles of a plasma react to the electric and magnetic fields of electromagnetic plasma waves. Two important consequences of wave–particle interactions are (a) particle energization, which heats the ions and/or electrons of a plasma and which can sometimes produce a new population of energetic particles, and (b) pitch-angle scattering, which changes the direction of a particles velocity vector with respect to the magnetic-field-line direction. Pitch-angle scattering allows some particles to overcome the mirror force such that the particles are “scattered” into the atmosphere and lost from the magnetosphere.

Finally, the *ionospheric outflow* of ions (Welling et al. 2015) is very important. The ionosphere (the ionized upper atmosphere) is a huge reservoir of cold ions that are gravitationally bound to the Earth. Various mechanisms can act to push ions upward out of the ionosphere along the magnetic-field lines into the magnetosphere.

## The Particle Populations of the Magnetosphere: The Subsystems

In one view of the magnetosphere as a system driven by the solar wind, the different particle populations of the magnetosphere can be considered as the interacting subsystems that make up the system. Of the 14 subsystems discussed in this section and listed in Table 2, the first 12 are ionized-particle populations and the last 2 are neutral-particle populations.

**Table 2:** Major particle populations (subsystems) in the magnetosphere; parameters of the populations can be found in Fig. 2

Plasma population	What and where	Origin	Roles in the system
Ionosphere	Ionized upper atmosphere	Sunlight on atmosphere and electron impact on atmosphere	Supplies plasma to magnetosphere Source of the plasmasphere and warm plasma cloak Dissipates system energy by Joule heating Hinders magnetospheric convection
Magnetosheath	Shocked solar-wind plasma bathing the outer surface of the magnetosphere	Solar wind	Plasma properties govern dayside reconnection rate Supplies solar-wind-origin plasma Transfers momentum into magnetosphere Generates ULF waves
Plasmasphere	Cold ionospheric-origin plasma in the dipolar region	Ionosphere	Provides home for whistler-mode hiss waves Provides home for EMIC waves Feeds drainage plume Feeds back on dayside reconnection
Ion plasma sheet (includes ring current)	Hot ions in magnetotail and dipolar region	Mantle, LLBL, Ionosphere	Diamagnetically distorts magnetic field Drives EMIC waves in dipole Can mass load dayside reconnection rate
Electron plasma sheet	Hot electrons in magnetotail and dipolar region	Solar wind	Home of the aurora and auroral currents Provides enhanced ionization of ionosphere Drives chorus waves May control ionospheric outflows to the warm plasma cloak Seed population for substorm-injected electrons
Electron radiation belt	Relativistic electrons in the dipolar region	Substorm-injected electrons	Energy sink for plasma waves Spacecraft damage Alters atmospheric chemistry
Ion radiation belt	Very energetic protons in dipolar region	Neutron decay, Solar wind Ion plasma sheet	not much is known about the origin and interactions of the outer proton belt
Substorm-injected electrons	Energetic electrons in the dipolar region	Electron plasma sheet	Drives whistler-mode chorus waves Seed population for electron radiation belt
Warm plasma cloak	Cool oxygen-rich plasma in dipolar region	Ionosphere	Mass loading of dayside reconnection rate Controls properties of ULF waves
Low-latitude boundary layer (LLBL)	Solar-wind plasma flowing tailward inside the magnetopause	Magnetosheath	Feeds plasma into the ion and electron plasma sheets
Mantle	Solar-wind plasma and cusp-ionosphere plasma moving into the magnetotail	Magnetosheath	Feeds plasma into the ion and electron plasma sheets Impacts reconnection rate in magnetotail
Plasmaspheric drainage plume	Cold plasma flowing from plasmasphere to dayside magnetopause	Plasmasphere	Loss of plasmasphere Mass loading of dayside reconnection rate Home of EMIC and hiss waves at larger radii than plasmasphere
Atmosphere	Neutral gas gravitationally bound to the Earth		Absorbs electrons and ions from the magnetosphere
Hydrogen geocorona	Neutral hydrogen evaporating off of atmosphere	Atmosphere	Causes charge-exchange loss of ion-plasma-sheet ions Has a charge-exchange interaction with the plasmasphere Is affected by magnetospheric energy input into upper atmosphere

EMIC: electromagnetic ion-cyclotron

There are two sources for the plasmas in the magnetosphere: (1) solar-wind plasma entering into the magnetosphere across the magnetopause and/or into the cusps and (2) outflow from the ionosphere into the magnetosphere. Solar-wind-origin plasma has an ionic composition like that of the solar wind, comprised of H^+^ and He^++^, while the ionospheric-origin plasma has an ionic composition of H^+^, He^+^, and O^+^. All of the plasmas have electrons. Excepting in very rare cases, plasmas are “charge neutral,” having equal amounts of positive and negative charges in a certain volume.

Note that the different plasmas of the magnetosphere can be geographically overlapping (colocated). Colocated plasmas have the opportunity to strongly interact with each other via plasma waves.

In Table 2, the major plasmas of the magnetosphere are outlined. In Fig. 2, the number density *n* and the temperature *T* of most of those plasmas are indicated schematically, each plasma exhibiting a range of *n* values and a range of *T* values. Note that these number densities are extremely low by terrestrial standards: the number density of molecules in the atmosphere at sea level is 2.5 × 10^19^ cm^−3^ and a very good vacuum in the laboratory is 3 × 10^9^ cm^−3^. When we speak here of high density and low density, it is relative to magnetospheric values. The temperatures T are measured in units of electron Volts (eV), with 1 eV = 11,600 K.

The first two plasmas discussed are boundaries of the magnetosphere that serve as plasma sources.

The *ionosphere* (Kelley 1989) is the ionized upper atmosphere beginning at a height of about 100 km above the ground. Most of the ionization comes from ultraviolet light from the Sun, but some of the ionization comes from the impact of energetic magnetospheric electrons onto the upper atmosphere. The ionosphere is more dense on the sunlit dayside of the Earth than it is on the nightside. The ionosphere is a major source of plasma for the magnetosphere, with magnetospheric activity affecting the rate of ion outflow. The outflow of cold electrons from the ionosphere (and of warmer photoelectrons from the atmosphere) is also important, allowing charge neutrality in the magnetosphere to be enforced. The ionosphere has its own set of rich phenomena that occur (e.g., instabilities, structuring, waves, flows; Hargreaves 1992; Blagoveshchenskii 2013): those ionospheric phenomena will not be considered here.

The *magnetosheath* (Kaymaz 1998) is the shocked solar-wind plasma that lies between the bow shock and the Earth’s magnetopause. As the solar-wind plasma is shocked in passing through the bow shock, the cool solar-wind plasma is converted into a hot and more-dense magnetosheath plasma that flows around the magnetosphere. The magnetosheath is the solar-wind plasma that makes contact with and leaks into the magnetosphere.

The *plasmasphere* (Darrouzet et al. 2009) is a cold dense plasma in the dipolar region of the magnetosphere. The plasmasphere is of ionospheric origin, caused by sunlit-driven outflow of cold ions from the dayside ionosphere. Near the Earth the plasmasphere corotates with the Earth. The properties of the dense plasmasphere largely control which plasma waves can exist in this geographic region of the magnetosphere; consequently, the plasmasphere controls a lot of the interactions between other plasmas. For a geographic sketch of the location of the plasmasphere in the magnetosphere, see Fig. 1 of Borovsky (2014a).

The *ion plasma sheet* (Denton and Borovsky 2009) is a hot low-density plasma, typically of solar-wind origin, but becoming rich in O^+^ ions from the ionosphere when magnetospheric activity is high. Two likely pathways for the solar-wind plasma to enter into the magnetotail to form the ion plasma sheet are via the cusp–mantle and via the low-latitude boundary layer. The ion plasma sheet flows from the magnetotail into the nightside outer-dipolar region following magnetic-field convection. As the ion plasma sheet flows into the dipole magnetic field, magnetic drifts become important for those hot ions and the flow deviates strongly from the magnetic-field convection pattern. An important loss mechanism for the ion plasma sheet is charge exchange with the hydrogen geocorona; of the ion-plasma-sheet ions that do not charge exchange, most flow out the dayside of the magnetosphere, but some of the hot ions can be trapped in the dipole magnetic field. The ion plasma sheet is synonymous with the “ring current” and the “partial ring current.” The plasma pressure of the ion plasma sheet can produce significant internal distortions to the dipole magnetic field of the magnetosphere. The ion plasma sheet is an important free energy source for plasma waves. The buildup of ionospheric oxygen in the ion plasma sheet can give the plasma sheet a high mass density: at those times the ion plasma sheet can alter the rate of reconnection on the dayside magnetopause. We will discuss the electrons of the plasma sheet separately from the ions of the plasma sheet, denoting them as two separate plasma populations: in the magnetotail they are colocated, but in the dipolar portions of the magnetosphere they have different convection paths. For a geographic sketch of the location of the ion plasma sheet in the magnetosphere, see Fig. 1 of Borovsky et al. (1997).

The *electron plasma sheet* (Elphic et al. 1999) is a hot low-density population of electrons that begins in the magnetotail like the ion plasma sheet. The electron plasma sheet convects sunward from the magnetotail into the outer dipole carried by magnetic-field convection. Unlike the hotter ions, the electron-plasma-sheet electrons follow the magnetic-field convection in the dipolar region. In the dipolar region, the plasma-sheet electrons convect toward the dayside magnetopause, where they will be lost from the system. On the way to the dayside magnetopause, the electrons suffer heavy pitch-angle-scattering losses to the atmosphere as they pass through the dipolar region. The electron plasma sheet is a free energy source for plasma waves, it is the electron source for substorm-injected electrons, and it is the magnetospheric location of the aurora. The warm plasma cloak is colocated with the electron plasma sheet in the dipolar region of the magnetosphere; it is likely that the electron plasma sheet (perhaps via auroral processes) drives the ionospheric outflows that produce the warm plasma cloak. For a geographic sketch of the location of the electron plasma sheet in the magnetosphere, see Fig. 5 of Thomsen et al. (1998).

The *electron radiation belt* (Friedel et al. 2002) is a population of very-high-energy (even relativistic) electrons orbiting in the dipolar portion of the magnetosphere. The electron radiation belt is divided into an inner belt close to the Earth and an outer belt further away. The seed population for the electron radiation belt is probably substorm-injected electrons. The radiation-belt electrons are energized by electromagnetic plasma waves in the dipolar magnetosphere. There are two major loss mechanisms: (1) pitch-angle scattering by plasma waves into the Earth’s atmosphere and (2) outward diffusion to the magnetopause. Intensifications of the radiation belt pose a hazard to spacecraft that are orbiting the Earth. The precipitation of radiation-belt electrons into the atmosphere can impact the chemistry of the middle atmosphere (NO_x_, HO_x_, and ozone) and the ionization and electrical conductivity of the middle atmosphere. For a geographic sketch of the location of the electron radiation belt in the magnetosphere, see Fig. 9 of Lyons et al. (1972).

The *ion radiation belt* (Borovsky et al. 2016) is a population of very-high-energy protons orbiting in the dipolar portion of the magnetosphere. Much less is known about the dynamics of the ion radiation belt than is known about the electron belt. There are two known sources for the ion radiation belt: (1) the decay of neutrons off the top of the atmosphere into protons and electrons and (2) the capture of energetic protons from the solar wind by the magnetosphere. A third potential source is the ion plasma sheet, particularly after substorm energization (Birn et al. 1997). For a geographic sketch of the location of the proton radiation belt in the magnetosphere, see Plate 1 of Beutier et al. (1995).

*Substorm-injected energetic electrons* (Birn et al. 1998) are energetic electrons delivered from the magnetotail into the dipolar region by substorms. A substorm is a brief interval (~ 30 min) of very intense earthward convection of magnetic flux from the magnetotail into the nightside dipolar region. As magnetospheric convection brings plasma earthward from the tail, the plasma is squeezed and energized. Normally, the hottest electrons of the magnetotail electron plasma sheet do not convect into the dipole owing to the dominance of magnetic drifts on the energetic electrons. However, substorm convection is unusually strong and the substorm can deliver a surge of those hot electrons into the dipole before the magnetic drifts affect them. These substorm-injected energetic electrons provide free energy for the growth of waves and probably serve as the seed population for the electron radiation belt. Substorm-injected electrons reside in the dipolar region for only a few hours after a substorm occurs, eventually being pitch-angle scattered into the atmosphere where they are lost. The precipitation of substorm-injected electrons into the atmosphere affects ionospheric conductivity and affects atmospheric chemistry and ionization.

The *warm plasma cloak* (Chappell et al. 2008) is an oxygen-rich population of 10s of eV ions (warmer than the plasmasphere) seen throughout the outer portions of the dipolar magnetosphere colocated with the electron-plasma-sheet population. The origin of the cloak is the ionosphere. Very little survey work has been done to characterize the warm plasma cloak and its time-varying properties. The warm plasma cloak influences the properties of plasma waves in the dipolar magnetosphere, particularly ULF waves. The high mass density of the cloak can have an influence on the dayside reconnection rate; hence, it may influence the amount of solar-wind driving of the magnetosphere. For a geographic sketch of the location of the warm plasma cloak in the magnetosphere, see Fig. 16 of Chappell et al. (2008).

The *low-latitude boundary layer* (Nemecek et al. 2015) is a mixture of hot magnetospheric plasma and hot magnetosheath plasma that resides just inside the magnetopause. It flows anti-sunward (in the direction of solar-wind flow) inside the magnetosphere. The low-latitude boundary layer is believed to be a pathway for solar-wind plasma (magnetosheath) to be delivered into the magnetotail to form the plasma sheet. For a geographic sketch of the location of the low-latitude boundary layer in the magnetosphere, see Fig. 3 of Reiff et al. (1994).

The *mantle* (Siscoe and Sanchez 1987) is a cool, anti-sunward-flowing plasma inside the magnetopause in the high-latitude (northern and southern) regions of the magnetosphere, particularly in the magnetotail. The mantle is magnetosheath plasma that enters into the magnetosphere in the northern and southern cusps. The mantle is another pathway for solar-wind plasma to enter into the magnetotail to form the plasma sheet. The mantle may also play a role in altering reconnection rates in the magnetotail (Hesse and Birn 2004). For a geographic sketch of the location of the mantle in the magnetosphere, see Fig. 1 of Eastwood et al. (2015).

The *plasmaspheric drainage plume* (Borovsky and Denton 2008) is a narrow band of cold, dense plasmaspheric plasma convecting sunward from the plasmasphere in the inner dipolar region to the dayside magnetopause, where it is lost from the magnetosphere. The drainage plume occurs during geomagnetic storms. The plume puts dense plasmaspheric plasma into the outer-dipolar regions; specific types of plasma waves can live in the dense drainage-plume plasma; and the plume allows those waves to exist in the outer-dipolar regions during storms. These waves in the plume have consequences for the evolution of the electron radiation belt. The dense plume plasma can also act to reduce the all-important dayside reconnection rate. For a geographic sketch of the location of the plasmaspheric drainage plume in the magnetosphere, see Fig. 1 of Borovsky (2014a).

The Earth’s *atmosphere* (thermosphere and mesosphere; Turunen et al. 2009) acts to absorb electrons and ions from the magnetosphere that manage to hit it, removing them from the magnetospheric system. Neutral winds in the upper atmosphere can force convection in the ionosphere and can alter magnetosphere–ionosphere coupling (Richmond and Matsushita 1975; Lu et al. 1995). Two well-established impacts on the atmosphere are (1) energetic-electron precipitation from the electron radiation belt and from substorm-injected electrons affecting the chemistry of NO_x_, HO_x_, and ozone in the middle atmosphere (Rodger et al. 2010; Andersson et al. 2012; Verronen et al. 2013; Seppala et al. 2015) and (2) energetic-electron precipitation from the electron radiation belt affecting the ionization and electrical conductivity of the middle atmosphere (Rycroft et al. 2000; Rodger et al. 2007; Borovsky 2017a). The Joule heating of the ionosphere associated with magnetospheric activity produces temperature changes in the upper atmosphere which change the atmospheric height profile and change the upper-atmospheric neutral winds (Fuller-Rowell et al. 1994; Burns et al. 2004; Wang et al. 2008; Lei et al. 2010; Weimer et al. 2013).

The *neutral-hydrogen geocorona* (exosphere; Ostgaard et al. 2003) is not a plasma; rather, it is a cloud of neutral-hydrogen atoms evaporating off the top of the upper atmosphere into the magnetosphere. Ions of the magnetosphere can charge exchange in the cloud of neutral hydrogen, changing an energetic ion into a very-low-energy ion. The geocorona provides an important loss for ion-plasma-sheet ions. Because of an energy dependence to the charge-exchange cross section, and because ions of different energies drift deeper into the geocorona, the charge-exchange losses produced by the geocorona can lead to free energy in the ion plasma sheet that can drive plasma waves (Thomsen et al. 2017). Magnetospheric activity produces observed alterations in the radial profile of the geocorona via changes in the charge exchange with the activity-dependent plasmasphere and via changes in the thermospheric temperature driven by magnetospheric Joule heating (Bailey and Gruntman 2013; Qin et al. 2017; Kuwabara et al. 2017). For a images of the location of the hydrogen geocorona, see Fig. 2 of Kameda et al. (2017).

**Fig. 3:**
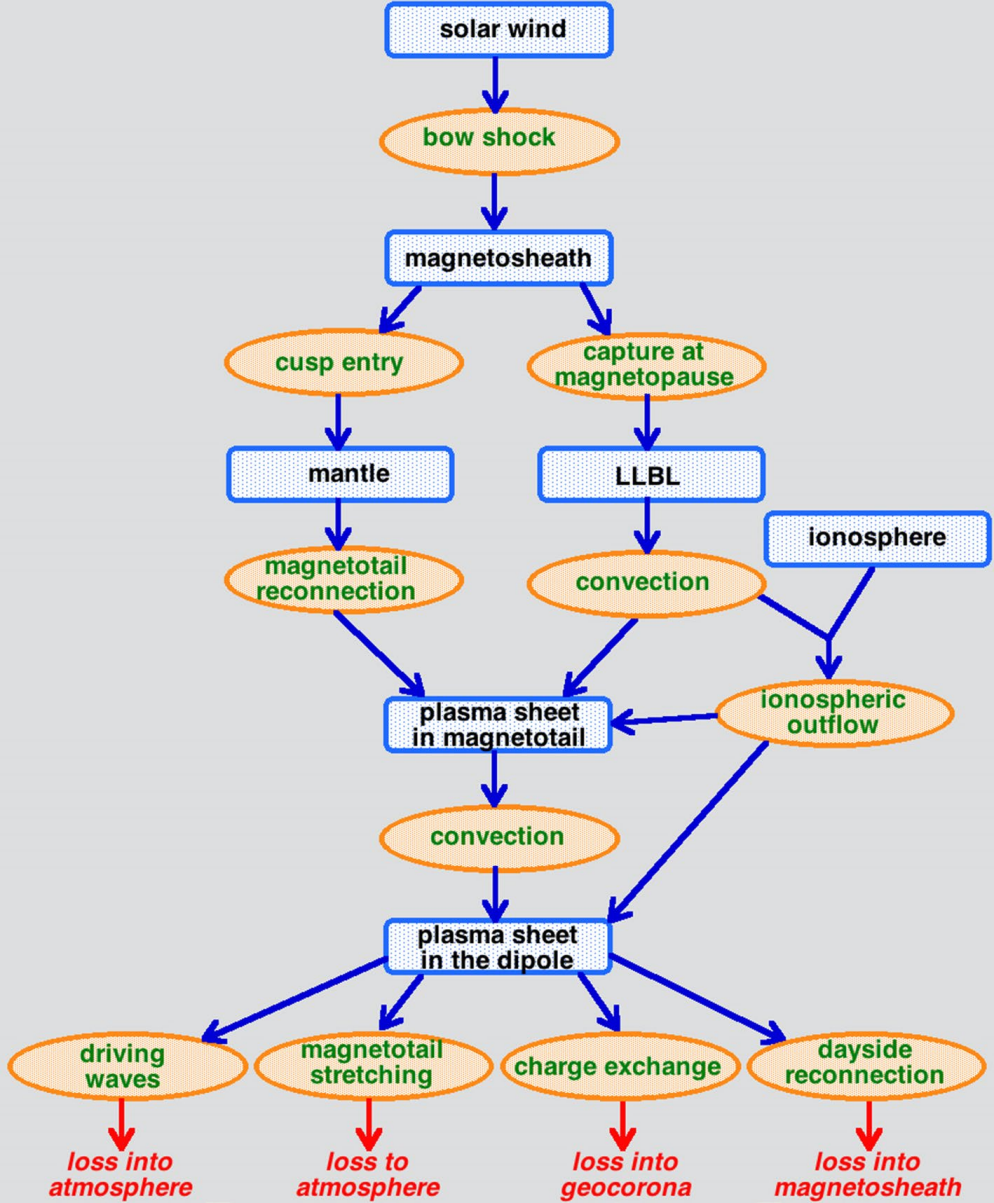
A sketch of the evolution of ion populations that eventually produce the ion plasma sheet, and the various ways in which the ion plasma sheet is lost from the magnetospheric system

In the third column of Table 2, the origins of each of the particle populations are listed. Each population is distinct in properties (cf. Fig. 2) and location, but in the magnetospheric system one population feeds another population with various physical mechanisms acting to transform the particles of one population into another. In general, the transformation involves heating (energization). (The exception to this heating is the production of the mantle from magnetosheath plasma.) To illustrate the transformation of populations, in Fig. 3 the evolutionary pathways of the ion populations that produce the ion plasma sheet are sketched, with the different particle populations represented as square boxes and physical processes represented as ovals. Starting at the top of the sketch, solar-wind plasma is transformed into magnetosheath plasma when the solar-wind plasma passes through the bow shock. The magnetosheath bathes the magnetosphere and can enter into the cusps to become the mantle plasma, and it can cross the magnetopause to become the low-latitude boundary layer (LLBL) plasma. The mantle plasma is transformed and enters into the ion plasma sheet in the magnetotail via the process of magnetic reconnection in the magnetotail; simultaneously, magnetospheric convection brings LLBL plasma into the magnetotail to join the ion plasma sheet. Additionally, if magnetospheric convection is strong, it leads to ion outflow from the ionosphere adding O^+^ ions to the ion plasma sheet. Magnetospheric convection delivers plasma sheet ions from the magnetotail into the nightside of the dipolar region of the magnetosphere. In the dipolar region, a number of loss mechanisms act, as noted at the bottom of the sketch of Fig. 3: the ion plasma sheet drives plasma waves that scatter its ions into the atmosphere, magnetotail stretching acts to scatter ions into the atmosphere, and charge-exchange collisions with the neutral-hydrogen geocorona remove the hot ion-plasma-sheet ions from the system. Finally, if the ions pass through the dipolar region to the dayside magnetopause, they are lost into the magnetosheath via dayside reconnection.

## Important Types of Plasma Waves and What They Do

Plasma waves are electromagnetic fluctuations in a plasma. The waves are characterized by frequency, by wavelength, and by the wave-vector direction with respect to the magnetic-field direction. Plasma waves can interact with the electrons in a plasma, with the ions in a plasma, or with the bulk parameters of the plasma such as density and velocity. Plasma waves do much of the coupling between the subsystems in the magnetosphere: typically one subsystem will create the waves and a second subsystem will be affected by the waves. In this sense, much of the plasma-wave coupling is “one way” with subsystem A affecting subsystem B, but not vice versa. Some of the most important types of plasma waves are collected into Table 3 and discussed below.

**Table 3:** Plasma-wave populations in the magnetospheric system

Type	Location	Properties	Typical period (s) or frequency (Hz)
ULF waves	Dipolar region	Driven by Kelvin–Helmholtz Driven by solar-wind compressions Driven by ion plasma sheet Produce radial diffusion of electron radiation belt Energize radiation belt	100–600 s
Electromagnetic ion-cyclotron waves (EMIC)	Dipolar region inside plasmasphere and plume	Driven by ion plasma sheet Scatter plasma-sheet ions and radiation-belt electrons into atmosphere	0.2–5 s
Whistler-mode chorus waves	Dipolar region outside of plasmasphere	Driven by substorm-injected electrons Energize the electron radiation belt Scatter plasma-sheet and radiation-belt electrons into atmosphere Produce diffuse aurora	100–5000 Hz
Whistler-mode hiss waves	Dipolar region inside plasmasphere and plume	Driven by electron plasma sheet? Scatter radiation-belt electrons into atmosphere	100–5000 Hz
Lightning-generated whistler waves	Dipolar region close to Earth	Associated with lightning occurrence Scatter radiation-belt electrons into atmosphere	100–10,000 Hz
Kelvin–Helmholtz oscillations	Magnetopause	Driven by magnetosheath flow Transport plasma from magnetosheath into magnetosphere Transport momentum into magnetosphere to produce magnetospheric convection Produce ULF waves in dipolar region	80–700 s
Magnetosonic waves (equatorial noise)	Dipolar region	Driven by ion plasma sheet Energize the electron radiation belt	20–150 Hz
Alfven waves	Throughout the magnetosphere	Initiate electrical currents that couple the motions of plasmas	60–500 s

*ULF waves* (Takahashi and Anderson 1992) are few-minute-period waves in the dipolar magnetosphere. They can be driven by a number of mechanisms: (1) by solar-wind-driven Kelvin–Helmholtz waves on the magnetopause, (2) by time-varying compressions of the dayside magnetosphere caused by time-varying properties of the solar-wind plasma, and (3) by free energy in the ion plasma sheet. ULF waves produce a radial diffusion of radiation-belt electrons and ions that redistribute the belt populations and can lead to loss of radiation-belt particles across the magnetopause. The redistribution also produces some energization of the radiation-belt electrons. The ULF waves mediate a coupling between the solar wind, the ion plasma sheet, and the ion and electron radiation belts.

*EMIC* (electromagnetic ion-cyclotron) waves (Usanova et al. 2012) are driven by free energy (such as thermal anisotropy) in the ion-plasma-sheet population. The dense plasmasphere and plume control where they can exist, and they are most prevalent where the ion plasma sheet and the plasmasphere/plume spatially overlap. EMIC waves are important for pitch-angle scattering ion-plasma-sheet ions and radiation-belt electrons into the atmosphere, removing them from the system. EMIC waves mediate a coupling between the ion plasma sheet and the electron radiation belt, with the plasmasphere influencing this coupling.

*Whistler-mode waves* (Stenzel 1999) are waves at audible frequencies that interact mainly with electrons. The whistler-wave activity is separated into two types: chorus (where individual temporal bursts of wave activity can be detected) and hiss (where wave activity is temporally continuous; Santolik and Chum 2009). (The reader can listen to the two types at http://www.astrosurf.com/luxorion/audiofiles-geomagnetosphere.htm). The local presence or absence of dense plasmaspheric plasma often determines which of the two types will be present.

*Whistler-mode chorus* waves (Meredith et al. 2003; Kasahara et al. 2018) are found in the dipolar region outside of the plasmasphere and drainage plume. These waves are driven by free energy in the electron-plasma-sheet population and the substorm-injected-electron population. They are particularly robust after a substorm occurs. Whistler-mode chorus waves are important for energizing radiation-belt electrons and for pitch-angle scattering radiation-belt electrons and plasma-sheet electrons into the atmosphere, removing them from the system. The whistler-mode scattering of electrons into the atmosphere produces the “diffuse aurora.” Whistler-mode chorus mediates a coupling between the electron plasma sheet and the electron radiation belt, with the plasmasphere influencing this coupling.

*Whistler-mode hiss* (Bortnik et al. 2011) is found in the dipolar region inside of the plasmasphere and drainage plume. At present, it is a controversy whether (a) whistler-mode hiss waves inside the plasmasphere are the result of whistler-mode chorus waves that propagate into the plasmasphere from outside or (b) whistler-mode hiss waves are directly driven by energetic electrons. Whistler-mode hiss is important for pitch-angle scattering radiation-belt electrons into the atmosphere. Whistler-mode hiss mediates a coupling between the plasmasphere and the electron radiation belt, with the electron plasma sheet playing a role in the coupling.

*Lightning-generated whistlers* (Oike et al. 2014) are found in the dipolar region very close to Earth. Their presence in the magnetosphere is associated with the intensity of thunderstorm activity in the atmosphere. Scattering of radiation-belt electrons into the atmosphere by lightning-generated whistler waves is a minor contributor to radiation-belt loss (Rodger et al. 2003).

*Kelvin–Helmholtz waves* (Nykyri and Otto 2001) are low-frequency propagating ripples on the magnetopause driven by the wind shear between the strongly flowing magnetosheath plasma and the slowly flowing magnetospheric plasma. They are akin to the ripples on a flag in the wind. Kelvin–Helmholtz waves are important for transporting magnetosheath plasma into the magnetosphere and for transporting momentum into the magnetosphere. The Kelvin–Helmholtz waves on the magnetopause also drive ULF waves inside the dipolar region of the magnetosphere, with those ULF waves affecting the radiation belts.

*Magnetosonic waves* (also known as “equatorial noise”; Santolik et al. 2016; Min et al. 2018) are medium-frequency waves in the dipolar region driven by free energy in the ion plasma sheet, with the waves intensifying when a substorm occurs. Magnetosonic waves interact with radiation-belt electrons to energize those electrons.

*Alfven waves* (Keiling 2009) are low-frequency electromagnetic waves whose propagation is ducted (focused) along the magnetic-field lines of the magnetosphere. Alfven waves are very important for establishing magnetic-field-aligned electrical currents that transport energy and deliver electromagnetic forces long distances along the magnetic-field lines. Such current systems (initiated by Alfven waves (Goertz and Boswell 1979)) couple magnetospheric convection to the ionospheric convection (Lysak 1990) and, in the polar regions, couple the flowing magnetosheath plasma to the polar ionosphere (Wright 1996). The auroral currents are also initiated with Alfven waves propagating from the plasma sheet in the magnetosphere to the ionosphere (Vogt 2002).

## Types of Activity in the Magnetospheric System

Often when magnetospheric activity is discussed in the research literature, what is specifically meant is *geomagnetic activity* (Rostoker 1972) as measured by one of several geomagnetic indices that are readily available. Each geomagnetic index is constructed of measurements from ground-based magnetometers, and each index is a measure of the intensity of a particular magnetosphere–ionosphere electrical current system. Hence, an increase in geomagnetic activity is the intensification of some current system in the magnetosphere. Here we will go much further: we will describe the various aspects of magnetospheric activity, as listed in Table 4.

**Table 4:** Types of activity in the magnetospheric system

Type	Explanation
Geomagnetic activity	The intensification of one of several current systems in the magnetosphere that can be measured by ground-based magnetometers
Dayside reconnection rate	Creation of magnetic connection between solar-wind plasma and the magnetosphere. Highly time variable with solar-wind time variations. Controls the amount of driving of the magnetosphere by the solar wind
Magnetotail growth/polar-cap size	Dayside reconnection adds magnetic flux into the magnetotail increasing the magnetic energy of the magnetospheric system
Magnetospheric convection	Transport of magnetic flux and plasma from the magnetotail into the dipolar region and then to the dayside magnetopause
Ionospheric convection	Horizontal transport of plasma from the dayside of the Earth to the nightside over the polar cap with lower-latitude return flows to the dayside
Magnetotail stretching	Intensification and earthward expansion of a cross-tail electrical current as flux is loaded into the magnetotail and as magnetospheric convection intensifies
Substorm occurrence	Large-scale morphological instability of the magnetotail. Produces enhanced transport in magnetosphere, injects energetic particles into dipolar region, greatly increases energy dissipation, is driver of enhanced auroral currents and particle precipitation
Global sawtooth oscillations	Large-scale morphological instability of the entire magnetosphere, dayside as well as nightside
Auroral currents	Field-aligned currents flowing between the nightside magnetosphere and the nightside ionosphere. Important for Joule dissipation of electromechanical energy
Auroral particle precipitation	Dissipation of magnetospheric energy. Produces localized enhanced electrical conductivity of the ionosphere
Ionospheric outflow	Upflow of ions into the magnetosphere in the cusp and auroral regions. Essential to build magnetospheric plasma populations. Eventually impacts dayside reconnection rate
Ring-current enhancement	Diamagnetic distortion of the dipolar magnetosphere caused by the particle pressure of the ion plasma sheet as the plasma-sheet population intensifies and moves into the dipole
Radiation-belt dropout	Sudden weakening of the intensity of the electron radiation belt in the early phases of a storm. Temporally correlated with an increase in solar-wind ram pressure
Radiation-belt intensification	Slow energization of the electron radiation belt during intervals of sustained magnetospheric driving by the solar wind
Storm	A strong elevation of all measures of magnetospheric activity associated with a feature in the solar wind that produces very strong driving of the magnetosphere. Two major types of storms: coronal-mass-ejection-driven and high-speed-stream-driven
Calm before the storm	Prior to most high-speed-stream-driven storms, there is a few-day period of anomalously low magnetospheric activity caused by a feature in the solar wind prior to the high-speed stream

The *dayside reconnection rate* (Komar and Cassak 2016) is the rate at which the magnetic field of the moving solar-wind plasma becomes connected to the Earth’s magnetic field. In a sense, it is the number of magnetic-field lines per unit time in the solar wind that become connected to the Earth. The dayside reconnection rate largely controls the amount of solar-wind driving of the magnetospheric system, and so the dayside reconnection rate largely controls all forms of magnetospheric activity. The properties of the solar wind vary with time, so the reconnection rate varies with time, with great variations in solar-wind parameters producing great variations in the reconnection rate. A particularly fast variation is caused by the sensitivity of the reconnection rate to the orientation of the magnetic-field vector in the solar-wind plasma, which varies on timescales of minutes. Note that there are time lags (minutes to days) between the reconnection rate and the various responses of the magnetospheric system.

*Magnetotail growth* (Petrinec and Russell 1996) results from dayside reconnection allowing the flowing solar wind to pull magnetic-field lines from the dayside magnetosphere and lay them down into the magnetotail, increasing the cross-sectional size of this cylindrical tail of field lines that are connected to the Earth. The *polar-cap size* (Huang et al. 2009) is directly related to the amount of magnetic flux in the magnetotail. Magnetotail growth represents an accumulation of magnetic energy that can be released by magnetic-field-line reconnection occurring in the tail. Reconnection in the tail allows magnetic-field lines to be transferred sunward back to the dayside magnetosphere, setting up magnetospheric convection. When magnetotail growth becomes substantial, a global magnetic instability of the magnetotail can occur to produce a substorm.

*Magnetotail stretching* (Gvozdevsky and Sergeev 1996) occurs during magnetotail growth: as more magnetic flux is added to the tail, a dawn-to-dusk horizontal sheet of current across the center of the magnetotail (separating the north half from the south half) intensifies and this current sheet spreads earthward. As the current sheet moves into the nightside dipole, it changes the near-Earth magnetic-field morphology from dipole-shaped field lines into magnetotail-morphology field lines.

*Magnetospheric convection* (Tanaka 2007) is the result of the transport of magnetic flux over the poles from the dayside magnetosphere into the magnetotail and the return of that magnetic flux through the magnetosphere from the tail to the dayside. Plasma is transported along with this magnetic convection, and the plasma is compressed and heated as it is brought in from the magnetotail. Magnetospheric convection sets up global electrical current systems in the magnetosphere that distort the morphology of the magnetic field and that give rise to the aurora.

*Ionospheric convection* (Weimer 2005) follows magnetospheric convection. As magnetic-field lines are carried by the solar wind over the poles from the dayside to the nightside after reconnection on the dayside, the footprints of those magnetic-field lines where they connect to the Earth are pulled toward the magnetotail. This pulling sets up a global flow of the ionospheric plasma from the dayside toward the nightside in the Earth’s polar regions. Consistent with the flow of magnetic-field lines returning from the magnetotail through the dipolar region to the dayside, there is a return flow in the ionosphere at lower latitudes from the nightside to the dayside.

A *substorm* (McPherron et al. 1973) is a short-lived surge in earthward convection in the magnetotail accompanied by a global change in the magnetic morphology of the tail, representing a transfer of stored magnetic energy into flow kinetic energy and plasma heating. The morphology change in the near-Earth nightside region is a return to a dipolar magnetic-field configuration from a tail configuration. Substorms deliver energetic substorm-injected electrons into the dipolar region, produce great increases in plasma-wave activity, and produce great increases in auroral activity. Substorms can occur randomly (associated with randomly occurring intervals of enhanced solar-wind driving) or they can occur periodically with recurrence periods of about 3 h.

*Global sawtooth oscillations* (Borovsky 2004) are periodic (~ 3 h) events wherein there is a magnetic-field stretching that occurs all around the Earth (dayside and nightside) followed by a sudden substorm-like magnetic-morphology change all around the earth. Global sawtooth oscillations occur during some types of magnetic storms.

*Auroral currents* (Strangeway 2012) are electrical currents that flow along magnetic-field lines between the nightside magnetosphere and the high-latitude nightside ionosphere. These currents are associated with magnetospheric convection, and the intensity of the currents increases when solar-wind driving increases. There are also substantial increases in the auroral currents when a substorm occurs. The currents produce a Joule dissipation of energy in the ionosphere, with that energy being extracted from the magnetosphere.

*Auroral particle precipitation* (Newell et al. 2016) produces diverse forms of optical airglow caused by magnetospheric particles hitting the upper atmosphere. Some types of aurora (e.g., auroral arcs) are associated with electron acceleration in electrical current systems in the nightside magnetosphere, and some types (e.g., diffuse aurora) are associated with the pitch-angle scattering of magnetospheric electrons and protons into the atmosphere. The auroral particle precipitation is an important loss mechanism for plasma-sheet electrons and the precipitation produces important changes in the electrical conductivity of the ionosphere. When the driving of the magnetosphere by the solar wind increases, auroral airglow intensities increase.

The *ionospheric outflow* of ions (Welling et al. 2015), particularly O^+^, increases as the driving of the magnetosphere by the solar wind increases. Ionospheric outflow has different properties coming from different regions of the Earth: e.g., the polar caps, the sunlit dayside, the nightside auroral zone. Various mechanisms act to push the ionospheric ions upward against gravity to produce the outflows, and these mechanisms may be strengthened by ionospheric convection and by magnetospheric particles hitting the upper atmosphere.

*Ring-current enhancement* (Liemohn et al. 2001) is caused by an increase in the plasma-sheet pressure in the dipolar magnetosphere, which is caused by sustained strong levels of magnetospheric convection. The pressure of the plasma-sheet ions in the dipole is associated with a diamagnetic current that distorts the magnetic morphology of the dipolar region.

*Radiation-belt dropouts* (Onsager et al. 2007) are rapid reductions of the number of radiation-belt electrons in the dipolar region of the magnetosphere. The dropouts are associated with intervals when the solar-wind number density becomes unusually high. In a dropout, the number density of the outer portions of the electron radiation belt can drop by one or two orders of magnitude in a few hours. The dropouts tend to occur in the early phases of a storm. The dropouts usually last for a fraction of a day.

*Radiation-belt intensifications* (Blake et al. 1997) occur in the outer portions of the electron radiation belt during long-duration (days) intervals of high magnetospheric activity. The sustained driving of plasma waves in the magnetospheric system leads to a steady energization of radiation-belt electrons by wave–particle interactions, producing a hotter and hotter radiation belt day after day.

A *magnetospheric storm* (Gonzalez et al. 1994) is an interval (a few hours to several days) wherein greatly enhanced driving of the magnetosphere by the solar wind produces greatly enhanced activations of magnetospheric phenomena. Storms can be identified with large-scale features in the solar wind plasma: hence, two major types of storms are magnetic-cloud-driven storms and high-speed-stream-driven storms (Borovsky and Denton 2006). These two types of storms exhibit systematically different magnetospheric phenomena.

The *calm before the storm* (Borovsky and Steinberg 2006) is a few-day-long interval of extremely quiet magnetospheric activity that tends to occur just prior to the occurrence of a high-speed-stream-driven storm. The extended calm interval can precondition the magnetosphere for the upcoming storm.

## Emergent Phenomena

In this section, we will discuss four examples of emergent phenomena in the magnetosphere, listed in Table 5. Here we will take the definition of “emergence” to be: “Emergence is the phase when new organizations and functions arise from the interactions of smaller, less complicated entities” (Mobus and Kalton 2015, p. 504). The connection of these entities has new consequences for the working of the system.

**Table 5:** Four examples of emergent phenomena in the Earth’s magnetospheric system

Phenomenon	Directly interacting subsystems	Necessary magnetospheric activity
Auroral arcs	Ion plasma sheet Electron plasma sheet Ionosphere	Magnetospheric convection
Pulsating-aurora patches	Electron plasma sheet Ion plasma sheet Ionosphere	Substorms
Substorms	Electron plasma sheet Ion plasma sheet	Magnetotail growth
The electron radiation belt	Electron plasma sheet Ion plasma sheet Substorm-injected electrons Plasmasphere Magnetosheath Geocorona	Substorms

The first example is *auroral arcs* (Borovsky 1993). An auroral arc is a long (1000s of km) east–west aligned thin (1–10 km) vertical curtain of airglow in the high-latitude nightside upper atmosphere. The airglow is caused by the impact of magnetospheric electrons that are accelerated downward into the atmosphere along magnetic-field lines that connect into the electron plasma sheet. The arcs are spatially colocated with electrical currents that flow between the magnetosphere and the ionosphere. The auroral currents dissipate electrical energy in the ionosphere and the accelerated electrons deposit energy in the atmosphere, with all of the energy coming from the magnetosphere. The amount of power extracted from the magnetosphere by auroral arcs is significant and must have an impact on the evolution of the magnetotail and the evolution of the ion and electron-plasma-sheet populations. When the solar wind is not driving the magnetosphere, auroral arcs are weak to absent. When solar-wind driving increases, the intensity of the auroral arcs and auroral currents increases, with about a 1-h time lag from the driving. One of the major unknowns of magnetospheric physics is the identity of the physical mechanisms in the magnetosphere that produce auroral arcs: it is likely that arcs are associated with magnetospheric convection and with the evolution of the plasma sheet ions and electrons in that convection. In order for auroral arcs to occur, several things must happen in the magnetospheric system. (1) Dayside reconnection must occur to enable the solar wind to load magnetic flux into the magnetotail. (2) Magnetospheric convection must occur and the electron and ion plasma sheets must evolve to create free energy to drive auroral electrical currents. (3) Some mechanism in the magnetosphere must act to drive the electrical current from the electron plasma sheet into the ionosphere. (4) Some mechanism must act to extract power from the ion plasma sheet and/or the electron plasma sheet to supply the auroral current system. (5) Some mechanism in the current system must act to accelerate the magnetospheric electrons into the atmosphere. (6) There must be a conducting ionosphere to close the electrical current, whose conductivity is enhanced locally by the impact of the electrons, probably nonlinearly.

The second example of an emergent phenomenon is *pulsating-aurora patches* (Jaynes 2018). Pulsating aurora appear in the high-latitude upper atmosphere after a substorm occurs. To an observer on the ground, the pulsating aurora appear as a set of optically blinking horizontal patches in the sky (blink periods ~ 1 s) at an altitude of about 100 km, slowly drifting horizontally relative to the ground observer. The patches each blink on and off periodically, with the individual patches blinking independently (Scourfield et al. 1972) and with different blink rates (Nishiyama et al. 2012). Typical patch horizontal sizes are 10s to 100s of km in the upper atmosphere (Royrvik and Davis 1977), with a tendency to be east–west elongated, with a patch lifetime of up to 10s of minutes (Grono et al. 2017). It is currently believed that the pulsating aurora is caused by the pitch-angle scattering of plasma-sheet electrons into the upper atmosphere by high-frequency (~ kHz) whistler-mode-chorus waves in the dipolar region of the magnetosphere outside of the plasmasphere. Unknowns are (a) what determines the shape of the patches, (b) what governs the drift of the patches, and (c) why the pitch-angle scattering turns on and off with a few-second periodicity. A number of things have to happen in the system for pulsating aurora to occur. (1) There must be an electron plasma sheet. (2) A substorm must occur to put free energy into the electron plasma sheet for the chorus waves to grow. (3) There must be pitch-angle scattering of the electrons by the waves. (4) Something must act to turn the wave scattering on and off, perhaps lower-frequency waves (such as ULF waves or magnetosonic waves) that are driven by free energy in the ion plasma sheet. Spatial structure in cool plasma such as the warm plasma cloak may play a role in shaping the pulsating patches. The pulsating-aurora patches are the visualizations of “coherent structures” (Cross and Hohenberg 1993; Shalizi et al. 2006; Dawes 2010) that arise in the very-large-scale electron plasma sheet.

A third example of an emergent phenomenon is a *substorm* (McPherron et al. 1973). Substorms are associated with a slow buildup of magnetic energy in the magnetotail followed by a rapid reduction in that built-up magnetic energy as the substorm initiates. Substorms have been described as a global plasma instability of the magnetotail triggered by the onset of magnetic-field-line reconnection in the magnetotail when the cross-tail current sheet becomes too thin (Birn et al. 2004), with reconnection allowing the magnetotail to morphologically change into a lower energy state. Substorm occurrence has also been described as a consequence of self-organized criticality in the magnetotail (Klimas et al. 2000). In this case, it has been postulated that there is a continuous distribution of localized dissipation events, both in time and in space, some of which can sometimes naturally self-organize into a more global event (Uritsky et al. 2002). For a substorm to occur, several things must be happening in the system. (1) Dayside reconnection must occur. (2) The solar wind must carry the newly reconnected field lines over the poles of the Earth into the magnetotail. (3) There must be plasma in the magnetotail to enable a cross-tail current sheet to form to stabilize the magnetotail as it grows. (4) There must be plasma in the magnetotail to enable magnetic-field-line reconnection to suddenly disrupt the current sheet allowing a sudden release of magnetic energy. (5) Something (unknown at present) must act to stop the magnetic-field-line reconnection to terminate the substorm. Another related emergent phenomena may be the sudden appearance of energetic substorm-injected electrons in the dipolar region: those energetic electrons having new consequences for the system.

**Fig. 4:**
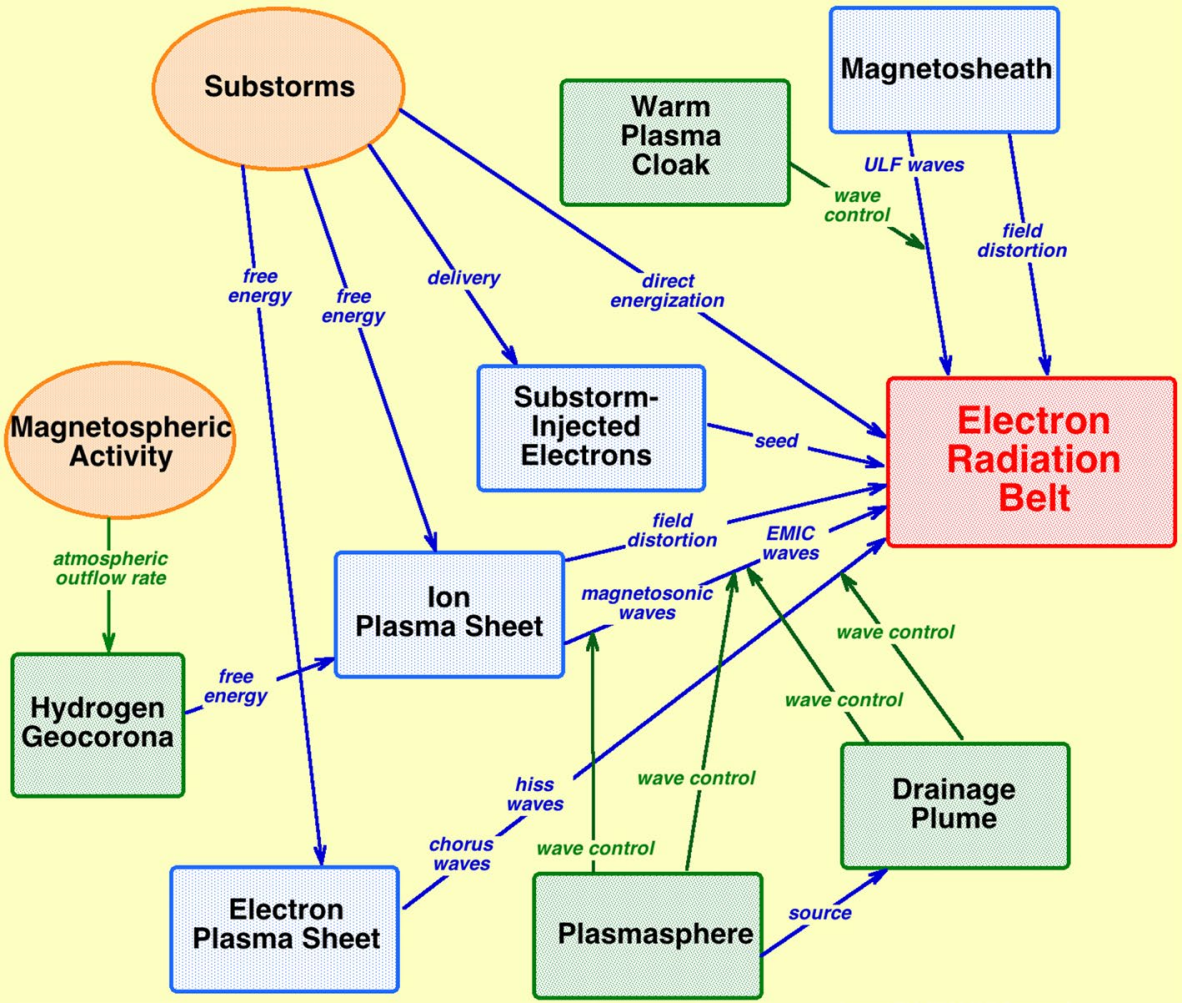
A sketch of the connections needed to create and evolve the electron radiation belt (red box). Not included in the sketch are magnetic-field distortions by the formation of cross-tail current and the rapid distortion of the magnetosphere by interplanetary shock waves

Fourth, the *electron radiation belt* (Thorne 2010) is probably the best example of an emergent phenomenon in the magnetospheric system, and it is itself an entire subsystem that emerges. If the radiation belt would not have been the first part of the magnetosphere to be discovered at the onset of the space age (e.g., Stern 1996), it would be difficult to predict its existence from even a modern reductionist understanding of the workings of the subsystems of the magnetospheric system. A great deal has to happen in the system to create the electron radiation belt (see also Fig. 4). (1) There must be (a) a dipolar portion to the magnetosphere so that very-high-energy electrons created can be trapped and (b) a magnetotail that can become globally unstable. (2) Energetic substorm-injected electrons must be repeatedly delivered into the dipolar magnetosphere by substorms to produce the seed-electron population for the radiation belt. (3) Substorms must continuously occur to put free energy into the electron plasma sheet to drive whistler-mode chorus waves. (4) Via wave–particle interactions the whistler-mode chorus waves must energize the substorm-injected electrons to very high energies. For the electron radiation belt to evolve, further processes must happen. (5) ULF waves must be created and the ULF waves must act to redistribute the radiation-belt via radial diffusion. (6) Whistler-mode hiss waves inside the plasmasphere must pitch-angle scatter the radiation-belt electrons into the atmosphere when the plasmasphere grows in radius during quiet magnetospheric activity. (7) ULF waves must radially diffuse the radiation-belt electrons outward during the early phases of storms to lose the electrons to the magnetopause to produce radiation-belt dropouts. (8) EMIC waves driven by the ion plasma sheet inside the plasmaspheric drainage plume must pitch-angle scatter the radiation-belt electrons into the atmosphere during the early phases of storms to help produce radiation-belt dropouts. Other system phenomena that are not depicted in Fig. 4 also play roles in the evolution of the electron radiation belt, including (a) magnetotail stretching, which distorts the dipole magnetic field and radially displaces the radiation belt, and (b) sudden distortions of the magnetic field by the passage of interplanetary shock waves in the solar wind, which is important for the evolution of the electron radiation belt close to the Earth. The electron radiation belt is born from substorm-injected electrons. If substorms are not occurring, there are no substorm-injected electrons in the dipolar region. After a substorm occurs, the injected electrons, with energies ~ 100 keV, have lifetimes of only a few hours before they are pitch-angle scattered into the atmosphere. When those electrons are energized (by chorus waves and by ULF processes) to ~ 1 MeV, they form a more-long-lived population and can reside in the outer-dipolar region for many days (Meredith et al. 2006) and in the inner dipolar region for years (Stassinopoulos and Verzariu 1971).

## The Applicability of System Descriptors to the Magnetosphere

Table 6 lists some of the systems science adjectives used to categorize and describe systems, and in Table 6 an assessment of each of those adjectives is given as it applies to the solar-wind-driven magnetosphere. An elaboration of that assessment is given in this section.

**Table 6:** Applicability of system descriptions to the magnetospheric system

Adjective	Applicable?	Statement
Adaptive	Yes	The magnetosphere reacts to and adapts to the time-varying solar-wind environments
Driven	Yes	By the time-varying solar wind, which transfers mass and energy into the system and drives a global circulation
Dissipative	Yes	Dissipation of electric currents, loss of particles to the atmosphere or to outside the system
Feedback	Yes	In response to strong reconnection there is mass loading of dayside reconnection by stored plasmas and by ionospheric outflows
Diverse	Yes	The subsystems (plasmas) of the magnetosphere are certainly diverse, it terms of their properties, origins, losses, evolutions, time and space locations, and interactions
Open	Yes	Energy and mass flow in from driver and flow out
Interconnected	Yes	Mediated by waves
Interdependent	Yes	Subsystems coevolve with each other
Emergence	Yes	4 examples: auroral arcs, pulsating aurora, substorms, the electron radiation belt
Nonlinear	Yes	There are nonlinear responses and feedback processes. There are multiple and variable time lags
Turbulent	Yes	Flow measurements in the magnetotail show clear evidence of flow turbulence
Cyclic behavior	Yes	The 3-hr substorm-recurrence period
Irreversible	Yes	Do not observe reverse convection, or de-energization of charged particles by plasma waves. The system has dissipation to the ionosphere and it has high-Reynolds-number regions
Criticality of self-organization	Yes	The conclusions of numerous data studies
Tipping point/phase transition	Yes	The substorm instability and the change in morphology of the magnetosphere
Complex	Yes	A removal of part of the magnetospheric system will change its behavior. The magnetosphere has diverse subsystems, multilevel interactions, multilevel structures, complicated interactions, and nonlinearities

*Adaptive* The magnetosphere adapts strongly to its solar-wind environment, changing as the properties of the solar wind vary. There are multiple time lags in the reaction of the subsystems to the solar wind, from minutes (reaction of the global system to changes in the solar-wind pressure) to a few days (intensification of the electron radiation belt in response to sustained solar-wind driving). Note that there is an informative historic comparison between the reaction of the magnetospheric system to driving by the solar wind and the reaction of the Earth’s atmospheric system to driving by the Sun, with the magnetosphere being labeled as a “compliant system” and the atmosphere labeled as a “persistent system” (Siscoe and Solomon 2006), the magnetosphere being very reactive (compliant) to the time-varying solar wind with the causes of magnetospheric storms clearly identifiable in the solar wind, whereas, on the contrary, there are no solar causes of hurricanes.

*Driven* A large number of statistical studies (e.g., Wygant et al. 1983; Newell et al. 2007, 2008; Borovsky and Denton 2014) have shown that the levels of the various measures of magnetospheric activity increase after certain parameters of the solar-wind change: this is perceived to be a driving of the system by the transport of mass and energy from the solar wind into the magnetosphere associated with the solar-wind control of the dayside reconnection rate.

*Dissipative* The magnetospheric system has direct dissipation of electrical currents in the resistive ionosphere (Weimer 2005), which damps magnetospheric convection and may extract thermal energy from the magnetosphere in driving the currents with pressure gradients. Loss of magnetospheric ions and electrons into the atmosphere is also a form of dissipation (Emery et al. 2008), as is ion and electron loss across the magnetopause, reducing the total thermal energy of the system. Similarly, the charge exchange of hot ions with the cold neutral-hydrogen geocorona also represents thermal energy loss (Kozyra et al. 2002).

**Fig. 5:**
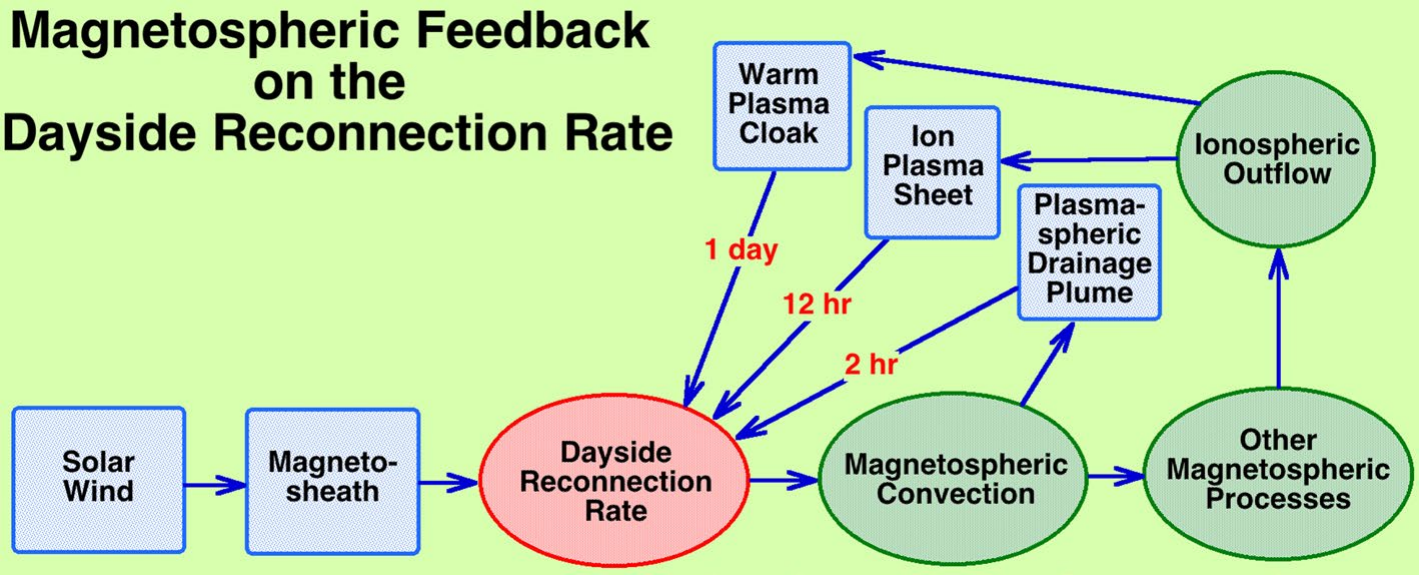
An influence diagram focusing on the feedback of high-mass-density magnetospheric plasma (the plasmaspheric drainage plume, the warm plasma cloak, and ionospheric oxygen in the ion plasma sheet) on the dayside reconnection rate. Indicated in red are the approximate lag times for the three plasmas to arrive at the dayside reconnection site after a change in the solar wind produces an increase in the reconnection rate

*Feedback* There are a couple of feedback loops in the magnetospheric system that can be identified. The first one (cf. Fig. 5) involves a feedback on solar-wind driving (Borovsky 2014a). (1) When the properties of the solar wind change to increase the dayside reconnection rate, (2) a stronger driving of the magnetosphere results, (3) this increases magnetospheric convection, and (4) the increased convection pulls some of the cold, dense, trapped plasmaspheric plasma out of the inner dipolar region to form a drainage plume. (5) The drainage plume convects to the dayside magnetosphere (6) where its high density acts to mass load the dayside reconnection rate, (7) reducing the rate at which the moving solar-wind plasma becomes magnetically connected to the Earth and reducing the driving of the magnetosphere by the solar wind. In a slightly different manner, the warm plasma cloak and the ion plasma sheet also partake in this feedback loop (Borovsky et al. 2013; cf. Fig. 5). A second feedback mechanism has been identified in computer simulations of the magnetosphere (Brambles et al. 2011; Ouellette et al. 2013). (1) At the start of a substorm, a new spot of reconnection occurs in the magnetotail near the Earth. (2) The enhanced substorm convection sends Poynting flux (Alfven waves) along the magnetic field from the reconnection site to the ionosphere. (3) The enhanced Poynting flux into the ionosphere causes a burst of O^+^ outflow from the ionosphere into the magnetosphere. (4) When the O^+^ reaches the reconnection site in the magnetotail, it mass loads the reconnection rate and shuts down the substorm.

*Diverse* See the discussion of the magnetosphere’s diverse subsystems in Sect. 5, those various subsystems having diverse origins and diverse properties, and interacting with each other in diverse manners.

*Open* As discussed in Sect. 6, mass (and energy) enters into the system from the solar wind (magnetosheath) and from the ionosphere. Mass (and energy) exits the system across the magnetopause, into the atmosphere, and by charge exchange with the geocorona.

*Interconnected* The diverse types of plasma waves that couple the various subsystems of the magnetosphere are discussed in Sect. 6.

*Interdependent* The various plasmas of the magnetosphere coevolve owing to their interactions. A prime example of this is the coevolution is the plasmasphere and the electron radiation belt. Chorus waves exist outside of the plasmasphere and chorus waves act to energize (intensify) the electron radiation belt; hiss waves live inside the plasmasphere and hiss waves act to scatter the radiation-belt electrons into the atmosphere. When magnetospheric convection is strong, the plasmasphere has a small radius; at those times, chorus waves throughout the dipolar region act to intensify the electron radiation belt (Pierrard and Benck 2012). When magnetospheric convection weakens, the plasmasphere grows in radius and nearly fills the dipolar region; at those times, hiss waves throughout the dipolar region scatter the radiation-belt electrons into the atmosphere (Borovsky and Denton 2009), weakening the electron radiation belt. A repeated pattern can be seen: the plasmasphere shrinks in size and the electron radiation belt intensifies, and the plasmasphere grows in size and the electron radiation belt weakens (Goldstein et al. 2005). It is likely that another pattern of coevolution is intensifications of the electron plasma sheet and intensifications of the warm plasma cloak, with the electron plasma sheet somehow acting to intensify the ionospheric outflows that give rise to the warm plasma cloak.

*Emergence* See the four examples of emergence in Table 5 and the discussion of Sect. 8: auroral arcs, pulsating aurora, substorms, and the electron radiation belt.

*Nonlinear* There are several identified nonlinearities in the magnetospheric system as driven by the solar wind. One is a storage-release reaction to solar-wind driving (Baker et al. 1997) wherein under steady solar-wind driving (steady dayside reconnection rate) the magnetotail slowly accumulates magnetic flux and magnetic energy and then suddenly releases the energy when a substorm instability occurs in the tail. A second observed nonlinearity in the reaction of the magnetosphere to driving by the solar wind is the polar-cap potential saturation that occurs during some magnetospheric storms: here the current systems inside the magnetosphere reach a saturation intensity even though solar-wind driving can be stronger and stronger. Various mechanisms for the saturation have been suggested (Borovsky et al. 2009), but there is no consensus on which mechanisms are acting. A third nonlinearity is a hysteresis-like effect called the atmospheric flywheel effect (Richmond and Matsushita 1975) wherein ionospheric convection remains to be driven when solar-wind driving shuts down owing to the inertia of the atmospheric convection pushing the ionosphere, where that atmospheric convection was itself driven by the previous solar-wind-driven ionospheric convection. A fourth example: the reaction of the electron radiation belt to solar-wind driving and to driving by magnetospheric processes is known to be dependent on the time-integrals of the processes (Borovsky 2017b).

*Turbulent* For a dynamical system, the presence of flow turbulence implies several aspects of complexity including unpredictability, irreproducibility, irreversibility, cross-scale coupling, enhanced dissipation, and mixing. Several examinations of the spacecraft-measured time series of the plasma-sheet velocity and magnetic field in the Earth’s magnetotail have found the flows to be turbulent (Borovsky et al. 1997; Voros et al. 2004; Stepanova et al. 2009), with large-eddy spatial scales of ~ 1 *R*_E_ (e.g., the tangled magnetic-field lines in Fig. 1). The plasma-sheet flows exhibit a classical turbulence power spectra of spatial scales (e.g., Kraichnan 1965; Boldyrev 2006), implying a cascade of energy from large scales to small scales.

*Cyclic behavior* The Earth’s magnetosphere exhibits cyclic behavior in its tendency for substorms (and global sawtooth oscillations) to recur with a ~ 3-h periodicity, that period being independent of substorm amplitude or phase of the solar cycle (Borovsky and Yakymenko 2017). What physically determines that the period should be 3 h in this storage-and-release cycle is not known. One idea about the cause of the substorm-occurrence periodicity is a feedback cycle with ionospheric outflow [Brambles et al. 2011; Ouellette et al. 2013] (cf. feedback discussion above/below), but see Lund et al. (2018) for contrary evidence. Note that other periodicities in magnetospheric activity associated with periodicities of the solar-wind driving can be seen: a 24-hr period associated with change in the amount of dipole tilt associated with the Earth’s rotation and a 27-day recurrence tendency for magnetospheric activity associated with the rotation period of the Sun (Denton et al. 2010).

*Irreversible* The magnetospheric system is irreversible for a number of reasons. First, the magnetosphere is dissipative (cf. the discussion above), with energy lost to the ionosphere, to the atmosphere, through the magnetopause, and via charge exchange. Second, the flows in the magnetotail plasma sheet are turbulent (see the discussion above); turbulent flows involve mixing and dissipation, making the flow irreversible. Third, the magnetospheric system is diffusive under the action of particle scattering by plasma waves (cf. Sect. 6). Fourth, under the action of magnetic reconnection plasma populations that become irreversibly mixed when they are magnetically connected (Gosling et al. 1990), which is irreversible. Finally, from an observer’s point of view, nothing close to a reversal of the global convection pattern in the magnetosphere has ever been seen: when the conditions of the solar wind are favorable for reconnection to add magnetic flux to the dayside magnetosphere rather than remove it (e.g., Lavraud et al. 2006), a new convection pattern in the magnetosphere arises rather than a reversal of the standard pattern (Weimer et al. 2010).

*Criticality of self-organizations* As discussed in Sects. 8 and 10, several studies have found evidence of self-organized criticality in the behavior of the magnetospheric system (Klimas et al. 2000; Uritsky et al. 2002; Valdivia et al. 2005, 2006, 2013).

*Tipping point/phase transition* The occurrence of substorms (described either by a global plasma instability or as a manifestation of self-organized criticality) is an example of a tipping point where stored energy is released and the dynamics of the system suddenly changes (cf. Lewis 1991; Sitnov et al. 2000).

*Complex* It is sometimes stated that a system is complex if it behavior is unpredictable and/or surprising (e.g., McDaniel and Driebe 2005). To assess whether or not the magnetosphere is a complex system, we will use two definitions of complex. First (Bar-Yam 1997): a system is complex if the removal of a part of the system changes its behavior. We could envision the effect of removing the ionosphere from the magnetospheric system. Among other effects, this would remove ionospheric outflow of ions, which would (1) prevent the occurrence of the plasmasphere and the drainage plume, which in turn would (2) greatly alter the populations of plasma waves that energize and evolve the electron radiation belt and would (3) eliminate the feedback loop that reduces solar-wind/magnetosphere coupling when the coupling gets too strong (cf. Fig. 5). In the magnetosphere, every plasma population directly affects at least one other population, and that in fact affects the entire system. By this first definition of complex, the magnetosphere is a complex system. For the second definition of complex (Lin et al. 2013), we will quote four sentences and add italics: “The key that distinguishes complex systems from simple systems is the different significance of interactions and connections among the subsystems or components. Generally, the components that make up a complex system *are not homogeneous* and *have multileveled structures*. There are not only interactions between the components but also *very complicated interactions among subsystems* and between levels. Especially, some of these interactions are *severely nonlinear*.” The magnetosphere is assessed against these statements, particularly the phrases marked in italics. (1) The components (subsystems here) of the magnetosphere are indeed diverse, *not homogeneous* (see Sect. 5, Table 2, and the discussion above). (2) There are *multilevel interactions* in the magnetosphere. An example is the ion plasma sheet simultaneously having microscopic and macroscopic interactions with the electron radiation belt (cf. Fig. 4). EMIC wave growth from free energy in the ion plasma sheet is a microscopic-level phenomena, as are wave–particle interactions when those waves pitch-angle scatter the electrons of the electron radiation belt (Thorne 2010); at the same time, the distortion of the magnetosphere’s magnetic dipole by the pressure of the ion plasma sheet (ring current) is a macroscopic-level phenomena, as is the alteration of the spatial location of the electron radiation belt by those global magnetic-field distortions (Kim and Chan 1997). Another example of multilevel structures is the fact that magnetic-field-line reconnection is initiated and proceeds via physics in the electron diffusion region of the reconnection site (Hesse et al. 1999), which has a scale size of ~ 1 km, but that reconnection allows the global reconfiguration of the 250,000-km-diameter magnetotail to a lower energy state (Birn and Hones 1981). A final example of multilevel structures is the plasma waves of the magnetosphere. The plasmas of the magnetosphere have nonzero temperatures (cf. Fig. 2) so the generation and propagation of the waves in the magnetosphere depend on the microphysical (kinetic distribution functions) properties of the plasmas. For instance, in the magnetotail non-Maxwellian ion and electron distribution functions are observed (Runov et al. 2015). The non-Maxwellian distributions suggest that some energization mechanism is operating, and this mechanism changes the nature of the plasma waves, affecting the way the waves accelerate and energize other populations of particles (Navarro et al. 2014). (3) The interactions within the magnetosphere are *very complicated*. An example (see Fig. 4) is the interaction between the electron plasma sheet and the electron radiation belt. This interaction involves free energy being created in the electron plasma sheet by the occurrence of substorms, allowing the electron plasma sheet to drive whistler-mode waves: the plasmasphere controls at what locations in the magnetosphere there will be whistler-mode chorus (which energizes the electron radiation belt) or whistler-mode hiss (which scatters the electron radiation belt into the atmosphere). Additionally, the electron plasma sheet is the seed population for the substorm-injected electrons that are in turn the seed population for the electron radiation belt. (4) There are several *nonlinearities* in the response of the magnetospheric system to the time-varying solar winds. One global nonlinearity is the feedback loop wherein the magnetosphere acts to reduce solar-wind driving when the driving gets too strong (see Fig. 5 and the feedback discussion above). A second global nonlinearity is polar-cap potential saturation (see the nonlinear discussion above), where some of the reactions of the magnetosphere to the solar wind appear to saturate. A third nonlinearity is the atmospheric flywheel effect (see the nonlinear discussion above). There are also mesoscale feedback loops between auroral currents and ionospheric conductivity: the auroral currents and particle precipitation modify the ionospheric conductivity, with the ionospheric conductivity then modifying the auroral currents (Ebihara and Tanaka 2018). Finally, there is certainly global nonlinearity in the storage-and release of magnetic energy in the magnetotail (Baker et al. 1997), released by the occurrence of substorms. This storage-and-release in fact leads to a well-known 3-hr periodicity to the reaction of the magnetosphere (periodic substorms or global sawtooth oscillations) to steady driving by the solar wind (Borovsky and Yakymenko 2017). A final example of nonlinearity is the interaction between storms and substorms (De Michelis et al. 2011), with storms being different because of substorms and substorm being different because of storms. Magnetospheric storms are associated with specific intervals of the solar wind that drive the magnetosphere to high levels of activity (Richardson et al. 2001; Siscoe and Solomon 2006). The intervals that drive the magnetosphere hard enough to produce a storm also drive substorms; hence, it would be unusual to have a storm without substorms. Storm phenomena would be different without substorms occurring. (A case in point is that substorms produce free energy for the driving of plasma waves that energize the electron radiation belt and substorms produce the seed electrons for the radiation belt (Su et al. 2014): without substorm occurrence, a storm probably would not produce a radiation-belt intensification.) Substorms are also different during storms owing to (a) the extreme distortion of the magnetosphere that occurs during a storm (Antonova 2002) and (b) the copious amounts of ionospheric plasma in the magnetosphere during a storm (Ono et al. 2010). By this second definition of complex, the magnetosphere is a complex system.

**Fig. 6:**
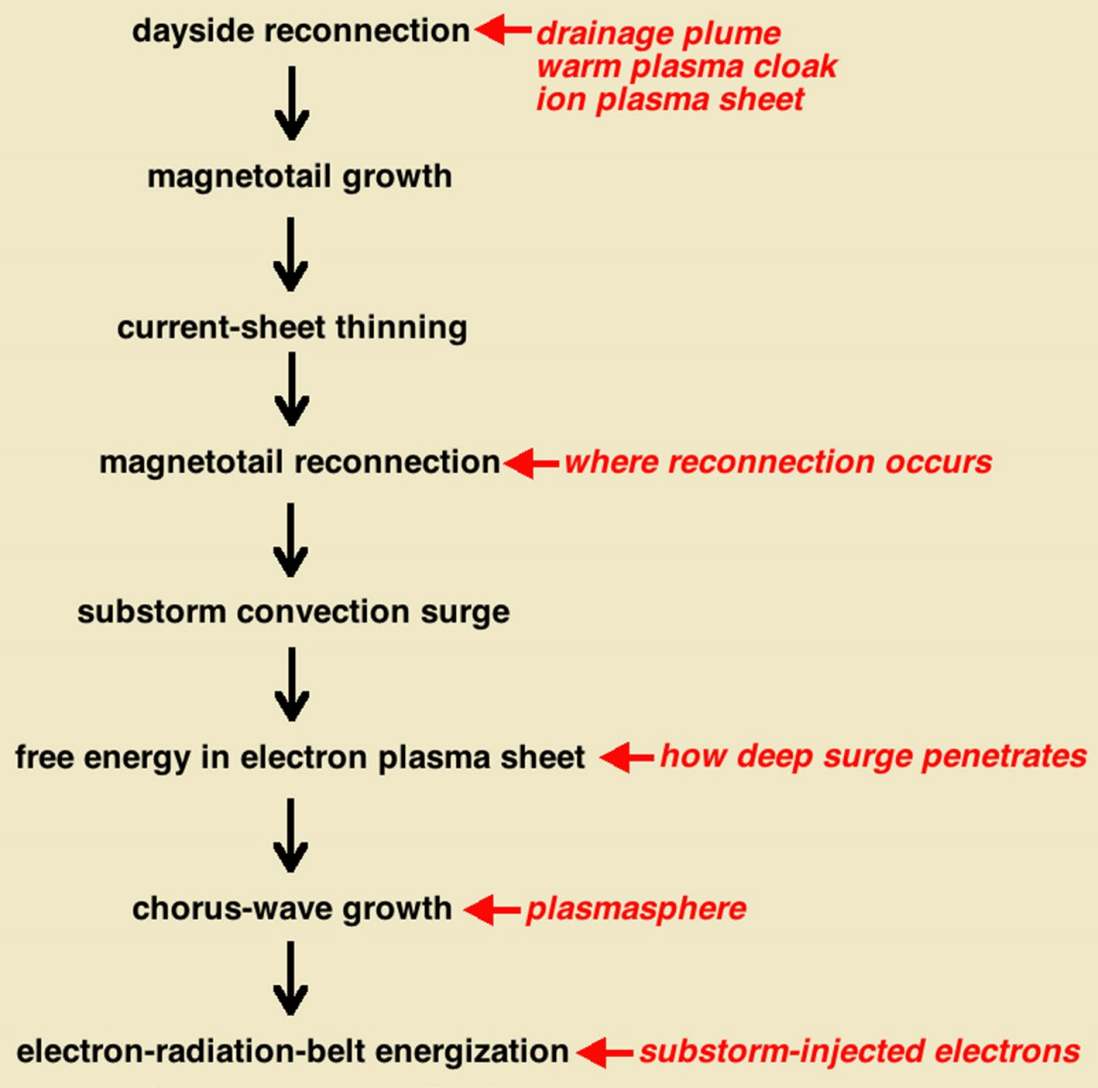
A causal chain of events from dayside reconnection to electron-radiation-belt energization by chorus waves is outlined in black. Some extenuating factors that influence that chain are noted in red

The magnetospheric system is certainly complicated, with long identifiable chain reactions wherein one thing gives rise to another which gives rise to yet another which gives rise etc., like Rube-Goldberg machines (Crease 2005). An example is sketched in Fig. 6, where dayside reconnection gives rise to the whistler-mode chorus waves that energize the electron radiation belt. [Compare the black portion of Fig. 6 with Fig. 1 of Crease (2005)]. Here the chain of reactions is outlined in black: dayside reconnection leads to magnetotail growth, which causes current-sheet thinning in the magnetotail, which leads to the onset of magnetotail reconnection, which produces a substorm convection surge earthward, which produces free energy in the electron-plasma-sheet population in the dipolar region, which causes whistler-mode chorus waves to grow, which energize radiation-belt electrons via wave–particle interactions. As an aside, note that the magnetosphere is a high-Reynolds-number fluid system, so that this chain of events is not entirely predictable or reproducible. But more importantly, this chain of events is also influenced by many other aspects of the magnetospheric system. Some of these are denoted in red in Fig. 6. From the top down, (a) the dayside reconnection rate is influenced by other plasmas in the magnetosphere, (b) where magnetotail reconnection occurs in the magnetotail depends on factors not yet understood, (c) how strong the convection surge is and how deep into the dipole it penetrates are controlled by factors not yet understood, and those strengths will determine whether free energy is given to the electron plasma sheet in the dipole. Continuing in Fig. 6, (d) where chorus waves grow depends on the state of the plasmasphere, and finally (e) how much energization the electron radiation belt obtains from the chorus waves depends on how many radiation-belt electrons were produced by substorm-injected electrons. Other linear chains could be drawn, such as dayside reconnection to the production of an auroral arc, or dayside reconnection to producing the warm plasma cloak (e.g., Fig. 5). But instead of being a simple set of linear chains of events, the operation of the magnetosphere is more like a tangle of Rube-Goldberg-like chains of events with the chains making multiple interactions and influencing each other. With these multiple connections, complicated moves to complex.

## Systems Science Work on the Magnetosphere

The behavior of the magnetospheric system impacts the behavior of the broader Earth system (Tinsley 2000; Georgieva et al. 2005; Rycroft et al. 2012; Sinnhuber et al. 2012; Clilverd et al. 2016; Lam and Tinsley 2016). Research on “Earth systems science” can be divided into two categories (cf. Rousseau 2017): (1) employing system-level thinking (e.g., accounting for connectivity, utilizing a diversity of expertise) and (2) applying the science of systems to the Earth. Magnetospheric systems science can also be described by these two categories. System-level thinking is well developed within the field of magnetospheric research with a lot of the connectivity of the system being understood and more connectivity being discovered. Some connections within the magnetospheric system that were recently realized are listed in Table 7.

**Table 7:** Recently uncovered connections in the magnetospheric system

Connection	References
Mass loading of dayside reconnection rate by high-mass-density magnetospheric plasma	Borovsky and Steinberg (2006)
Magnetosonic waves driven by ion plasma sheet can energize radiation-belt electrons	Horne et al. (2007)
Role of geocorona in giving free energy to ion plasma sheet to drive waves	Meredith et al. (2008)
Realization of the warm plasma cloak	Chappell et al. (2008)
Radiation-belt electron scattering by plasmaspheric drainage plumes	Summers et al. (2008)
Plasmaspheric hiss can come from whistler-mode chorus	Bortnik et al. (2008) Chum and Santolik (2005)
Atmosphere chemistry affected by magnetospheric electrons	Verronen et al. (2011)
Relation of auroral arcs to magnetotail stretching	Birn et al. (2012)
Role of warm plasma cloak in modifying ULF wave properties	Takahashi et al. (2014)
Radiation-belt electron acceleration by time-domain structures	Mozer et al. (2014)

Systems science work on the Earth’s magnetosphere driven by the time-dependent solar wind has been ongoing for decades, yet a treatment that accounts for all of the major subsystems and their interactions has not yet been attained.

In examining the behavior of the full, interconnected magnetospheric system, global physics-based simulation codes are probably the furthest along. The global-simulation codes solve the ideal-MHD equations in time on a three-dimensional grid, with a dipole magnetic field coming out of the Earth and the Earth’s surface covered by an electrically resistive ionosphere and with a supersonic solar-wind plasma blowing at the Earth (Gombosi et al. 2000; White et al. 2001; Raeder et al. 2001; Lyon et al. 2004). Evolving versions of the global-MHD simulation codes involve adding a non-MHD model in the dipolar region of the magnetosphere to account for some aspects of magnetic drifts (Toffoletto et al. 2004; Welling and Ridley 2010; Yu et al. 2016; Jordanova et al. 2018), adding a model of the cold plasmasphere in the dipolar region (Ouellette et al. 2016), and adding ionospheric outflow (Glocer et al. 2009a; Brambles et al. 2011). These global-MHD simulations have been successful at reproducing several aspects of the system behavior of the magnetosphere as driven by the solar wind. But note that even the most-advanced physics-based simulation codes still lack all of the magnetosphere’s plasma populations, still lack the correct inter-plasma coupling mechanisms, and do not capture all types of magnetospheric activity. Additionally, the codes do not capture the wide range of time and spatial scales involved.

Simulating a more-reduced portion of the magnetospheric system, physics-based simulations codes for the electron-radiation-belt evolution have been developed that focus on the physical mechanisms that are most important to the electron belt (Bourdarie et al. 1996; Shprits et al. 2008a, b; Glocer et al. 2009b; Reeves et al. 2012; Fok et al. 2014). [There are much-less-sophisticated simulation codes for the ion radiation belt (e.g., Boscher et al. 1998; Vacaresse et al. 1999; Panasyuk 2004).] These physics-based codes have been successful at reproducing the major evolutionary aspects of the electron radiation belt as driven by magnetospheric convection and have been very useful for Gedanken experiments to explore the relative importance of various physical mechanisms.

There have also been simplified mathematical models built to look at the minimal descriptions needed to reproduce characteristic system reactions (such as substorm recurrence and chaos) to solar-wind driving (Smith et al. 1986; Goertz and Smith 1989; Goertz et al. 1991, 1993; Vassiliadis et al. 1993; Klimas et al. 1996, 1997, 2004; Horton and Doxas 1998; Valdivia et al. 1996, 1999, 2003, 2006; Freeman and Morley 2004; Spencer et al. 2006).

Most systems-science-oriented data analysis of the magnetosphere has focused on one aspect of magnetospheric activity at a time, usually on geomagnetic activity as measured by a single geomagnetic index. The goal of the data analysis has been to determine and analyze characteristic system behaviors of the magnetosphere, including examinations of chaotic output (Shan et al. 1991a, b; Sharma et al. 1993; Pavlos et al. 1994; Vassiliadis et al. 1995; Ukhorskiy et al. 2003), examination of multifracticality (Consolini et al. 1996; Voros and Jankovicova 2002; Consolini and De Michelis 2011), looking at the recurrence statistics of substorm events (Borovsky et al. 1993; Prichard et al. 1996; Morley et al. 2009; Forsyth et al. 2015; Chu et al. 2015; Borovsky and Yakymenko 2017), substorm amplitudes (Borovsky and Nemzek 1994; Morley et al. 2007; Borovsky and Yakymenko 2017), and examining data for evidence of self-organized criticality (Uritsky et al. 2001; Crosby et al. 2005; Sharma et al. 2005, 2016). In a similar vein, there has also been work to look at the amplitude scaling of auroral-brightening events in the upper atmosphere (Lui et al. 2000; Lui 2002, 2004; Uritsky et al. 2002).

In the spirit of analyzing the holistic behavior of the multiconnected magnetospheric system, data analysis of the multivariable solar wind (represented by a solar-wind state vector) driving the multivariable magnetosphere (represented by a magnetospheric state vector) has been performed, although not yet accounting for all of the major subsystems of the magnetosphere. Fung and coworkers (Fung and Shao 2008; Fung et al. 2016) have looked at independently predicting each element of a magnetospheric state vector from the full solar-wind state vector and then assembling a predicted magnetospheric state vector from its individually predicted elements. Magnetospheric state vectors with up to 4 dimensions (i.e., 4 simultaneous measures of the magnetosphere) have been assembled in this manner. To account for the interconnectedness of the magnetospheric system, Borovsky and Denton (Borovsky 2014b, 2018; Borovsky and Denton 2014, 2018) looked at vector correlations between the time-dependent solar-wind state vector and the time-dependent magnetospheric state vector. This vector–vector correlation methodology reveals the simultaneous coupled reactions of multiple aspects of the magnetospheric system to driving by the solar wind. Magnetospheric state vectors involving up to 11 simultaneous magnetospheric measures (11 dimensions) have been utilized. Future studies using additional measures of the magnetosphere are planned.

Detailed descriptions of these and other systems science investigations of the Earth’s magnetosphere can be found in the reviews by Voros (1994), Lakhina (1994), Vassiliadis (2000, 2006), Chapman et al. (2004), Valdivia et al. (2005, 2013), Sharma (2010, 2014), Pavlos et al. (2011) and Stepanova and Valdivia (2016).

A holistic systems science treatment of the coupled magnetospheric system is lacking, i.e., there has been no systems science research on the magnetosphere that deals with the diversity of its interconnected subsystems and the complexities of its varied interactions. And there has been insufficient coordination of magnetospheric science with the broader Earth systems science. Systems science has the potential to (1) inform about the behavior of the magnetosphere in response to the solar wind, (2) to find new phenomena, (3) to uncover rules, (4) to find hidden connections and unrealized feedback, and (5) to predict what is important to measure. As the present review points out, the magnetospheric system is unique, with its diverse subsystems, the huge ranges of important spatial and temporal scales, the driving by the time-varying solar wind, and the one-way coupling by plasma waves. Indeed, the global physics-based simulation codes that are designed for the magnetosphere are unique. A question can be asked: will unique systems science tools need to be developed to study the magnetospheric system? And can studying the magnetospheric system (and developing those tools) advance the “science of systems”?

## Discussion: Critical Things That Are Not Known

In Sect. 1, it is pointed out that a motivation for the present paper is the statement of Lin et al. (2013): “when hoping to understand the behaviors of a complex system, one needs to analyze not only how different components work together to form the behaviors of the whole system, but also the behaviors of the individual parts. Without deep and specific comprehension of the behaviors of the individual parts, there will be no way to capture the behaviors of the complex system.” Further, in Sect. 10 the goal was stated of putting the entire magnetospheric system together to perform systems science on the entire integrated system. In hoping for that goal, one must unfortunately realize that not everything is known about the parts of the magnetospheric system, and probably not everything has been discovered.

At present, the field of magnetospheric physics is making great progress on understanding the underlying physical processes acting in the system (cf. Table 7), but the degree of maturity of the system understanding has not been assessed. From a systems science point of view, feedback phenomena are just being uncovered, coupling pathways and coupling mechanisms are still being explored, the multiple time lags in the system have yet to be surveyed, and large-scale systems simulations lack much of the relevant physics. Still, we believe that the progress in our understanding of how the different subsystems, and even levels within them, combined with the large amount of spatial and temporal data available in this magnetospheric system, provides a test bed to advance and test system sciences ideas, concepts, and techniques.

There are several identified outstanding unknowns about the Earth’s magnetosphere (cf. Denton et al. 2016). A sampling is the following. (1) Our knowledge of how the solar wind couples to the Earth’s magnetosphere is in general poor, with several fundamental basic-physics issues yet to be settled. (2) The mechanisms that manifest the inflow of plasma from the magnetosheath into the magnetosphere are poorly understood and what controls the amount of inflow is not known. (3) The physical mechanisms in the magnetosphere that extract energy and produce auroral arcs are not known. (4) The role of cold ions in the magnetosphere for controlling plasma waves is not fully understood, and the role of cold electrons in the magnetosphere for controlling plasma waves is very poorly understood. (5) Survey work is lacking that would tell us the general properties of the warm plasma cloak and that would give us a rudimentary understanding about its evolution. (6) To really understand the extent of wave–particle interactions among the plasmas of the magnetosphere, the time-varying properties of the plasma waves (intensities, frequency spectra, and distributions of wave-vector directions) need to be known everywhere (all radii, all latitudes, all longitudes), whereas there are no thorough surveys of these properties. Finally, (7) there is a lack of understanding of when, where, and by how much ionospheric ions flow out of the magnetosphere, a lack of understanding of the mechanisms producing the outflows, and a lack of understanding about what controls these outflows.
